# Expression of PKM2 in wound keratinocytes is coupled to angiogenesis during skin repair in vivo and in HaCaT keratinocytes in vitro

**DOI:** 10.1007/s00109-022-02280-6

**Published:** 2023-01-12

**Authors:** Khrystyna Sych, Simon P. Nold, Josef Pfeilschifter, Rajkumar Vutukuri, Jana Meisterknecht, Ilka Wittig, Stefan Frank, Itamar Goren

**Affiliations:** 1 Pharmazentrum Frankfurt/ZAFES, General Pharmacology and Toxicology, Faculty of Medicine, Goethe-University, Frankfurt, Frankfurt am Main, Germany; 2 Functional Proteomics, Institute for Cardiovascular Physiology, Goethe-University, Frankfurt, Theodor-Stern-Kai 7, D-60590 Frankfurt, Frankfurt am Main, Germany; 3 German Centre for Cardiovascular Research (DZHK), Partner Site Rhein-Main, Frankfurt am Main, Germany

**Keywords:** Wound healing, Metabolic cues, Warburg phenomenon, PKM2, Keratinocytes

## Abstract

**Abstract:**

An injured skin is rapidly restored in a manner of wound healing. We have previously shown that intact insulin signaling and glucose uptake are fundamental to proper wound closure. Consequently, under exacerbated inflammation, compromised insulin action and glucose uptake lead to impaired healing. However, in spite of the increased attention to cell metabolism during tissue regeneration, metabolic mediators that govern cellular and physiological processes throughout skin repair remained largely elusive. Through assessment of mRNA using real-time PCR and protein blot analysis, we report that healing of cutaneous wounds comprise a boosted expression of genes involved in glycolysis, oxidative phosphorylation, pentose phosphate shunt, and glutamine anaplerosis. We further focused on the functional role of pyruvate kinase M (PKM) isoenzymes that catalyze the final and rate-limiting step of glycolysis. Whereas the expression of the metabolic constitutively active Pkm1 isozyme remained almost unchanged, Pkm2 is augmented during the inflammatory phase of healing. The immunohistochemistry and RNA in situ hybridization analysis showed a confined Pkm2 expression to keratinocytes of the hyperproliferative epithelium and, to a lesser extent, infiltrating neutrophils and monocytes as well as later on in macrophages. Notably, the expression of Pkm2 in keratinocytes facing the wound bed side colocalized with VEGF expression. The in vitro knockdown of PKM2 in HaCaT keratinocytes using small interfering (si) RNA confirmed an acute role for PKM2 in facilitating the complete induction of VEGF mRNA and protein expression in keratinocytes; this function is mainly HIF-1α independent.

**Key messages:**

• Wound healing involves activation of glycolysis, oxidative phosphorylation, pentos-phosphate shunt, and replenishment of tri-carboxylic acid (TCA) cycle through glutamine anaplerosis.

• The pyruvate kinase M2 (PKM2) isoform is upregulated during the inflammatory phase of cutaneous healing, mainly in keratinocytes of hyperproliferative epithelia.

• In vivo, the expression of VEGF in wound keratinocytes is colocalized with PKM2.

• PKM2 is required for full induction of VEGF in HaCaT keratinocytes in vitro.

**Supplementary Information:**

The online version contains supplementary material available at 10.1007/s00109-022-02280-6.

## Introduction

Since the seminal work of Otto Warburg during the 1920s on ascites, the metabolic peculiarity of proliferating cells during pathological insults such as cancer and inflammation has been a matter of consistent scientific interest. Warburg’s observation that as opposed to resting cells, in rapidly growing tissues such as tumors, cells prefer to ferment glucose through the less efficient glycolysis pathway, even under aerobic conditions; an effect later coined the “Warburg phenomenon” [1]. Although this feature of fast-growing tumors to consume glucose and rapidly produce lactate was widely accepted, in the post-double helix era, recognizing that cancer is a genetic disease, researchers focused on genes rather than metabolites that promote malignancy. While Warburg’s conviction-aerobic fermentation may be caused by mitochondrial dysfunction turned out to be incorrect, Warburg’s view that metabolic processes drive malignancy [2] has revived [3, 4]. The notion that metabolic pathways could even determine the fate of tumorigenesis regained attention. Metabolites were proven to regulate oncogenic activity [5], as well as other pathophysiological processes like inflammation [6, 7] and immune response [8]. Similarly, it has emerged that metabolic conditions directly affect the outcome of cutaneous wound healing, an inflammatory-driven process that involves massive cell influx and outflow, proliferation, and remodeling. Accordingly, we have shown that intact insulin signaling and correct glucose uptake are essential to achieve wound closure [9, 10]. We proposed that sustained inflammation leads to shutting down insulin signaling in keratinocytes, also referred to as “insulin resistance” as manifested in obese mice [11]. Indeed, chronic energy surplus leads to adipose tissue expansion, and concomitant infiltration of inflammatory cells, in turn, induce a low-grade inflammation termed “metaflammation,” exacerbating the metabolic dysfunction [12]. In fact, we have shown that adipocytes exhibit a pro-inflammatory feature upon maturation in vitro, and contribute to healing impairment in obese mice [13]. Hence, given the central role of energy balance in the fate of cutaneous wound closure, we decided to journey into the metabolic landscape of normally healed wounds. Not surprisingly, skin injury induces the activation of the catabolic cascade of glycolysis as depicted by the expression of glyceraldehyde 3-phosphate dehydrogenase (Gapdh) and the glycolysis inducer, 6-phosphofructo-2-kinase/fructose-2,6-biphosphatase 3 (Pfkfb3), as well as mitochondrial oxidative phosphorylation (OXPHOS) as appeared from the expression of the mitochondria outer-membrane protein voltage-dependent anion channel 1 (Vdac1) and the mitochondrial-associated kinase hexokinase II (HkII). Induction of the anabolic pentose phosphate pathway (PPP) was also observed as manifested in gene expression of the first enzyme glucose-6-phosphate dehydrogenase (G6pdx), and the inducer of this shunt, the glycolytic phosphoglycerate mutase 1 (Pgam1). Glutamine anaplerosis of the mitochondrial tricarboxylic acid (TCA) cycle was also detected, as appears from the activation of the two pivotal genes that are involved: glutaminase (Gls) and glutamate dehydrogenase (Glud1). In this context, robust activation of glutamine synthase mRNA and protein, a hallmark for metabolically active tissue, was observed throughout the healing process. Of genes that were greatly induced during cutaneous repair and their product(s) primarily control metabolic processes, we were alerted to the temporal and spatial expression of the M2 isoform of pyruvate kinase (PK) PKM2. Pyruvate kinase is a homotetrameric enzyme that catalyzes the last and rate-limiting step of the glycolytic pathway, transferring the high-energy phosphate from phosphoenolpyruvate (PEP) to 2 ADP molecules, generating 2 ATP molecules and pyruvate. Two distinct genes, PKLR and PKM, code for PK in mammals. The PKLR gene codes for the L and R isoforms expressed in the liver and red blood cells, respectively, under the control of two different promoters [14]. Conversely, the PKM gene is expressed in the rest of the tissues and codes for the alternatively spliced exon 9 or exon 10 products, expressing variant M1 (PKM1) or variant M2 (PKM2), respectively [15]. PKM1 is a constitutively active tetrameric isoform, whereas PKM2 tetramerizes, and, hence, metabolic activation is allosterically regulated by metabolic cues such as the glycolytic intermediate fructose 1,6-bisphosphate (FBP) [16], amino acid serine [17], and the purine nucleotide synthesis intermediate SAICAR. Furthermore, PKM2 glycolytic activity is suppressed by growth signals directly through tyrosine phosphorylation of PKM2 [18], or indirectly by binding to a tyrosine-phosphorylated protein [19]. PKM1 is abundantly expressed in terminally differentiated and constantly high rate ATP-consuming tissues like muscles or neurons, whereas rapidly growing tissues like fetal tissues or tumors express mainly the regulated PKM2 isoform. This seeming disadvantageous expression of an enzyme having less pyruvate kinase activity can be explained by the existence of an alternative acceptor for the phosphate donated from PEP in the form of phosphoglycerate mutase (PGAM), thereby “rewiring” glycolysis and conveying glycolytic metabolite to replenish the metabolic demands of a growing tissue [20, 21]. A groundbreaking work of Christofk, Vander Heiden, Cantley, and their co-workers showed the significance of PKM2 expression for cancer metabolism and tumor growth [19]. Subsequently, the M2 isoform was considered as one of the key drivers of the Warburg phenomenon [22, 23]. Surfing into the ocean of PKM2 literature, a feature of multitasking, moonlighting protein has emerged. Though some of these features were revised [24], the metabolic requirements for PKM2 were confined to some tumors or stages of tumorigenesis [25, 26]. The parallelism of tumor biology and wound healing [27] motivated us to take a closer look at the role of Pkm2 isoforms in the murine model of skin excisional repair. Immunohistochemistry (IHC) staining revealed the expression of Pkm2 mainly confined to keratinocytes in the hyperproliferative epithelial tongue and, to a lesser extent, in infiltrating monocytes/macrophages. Additionally, we observed the co-localization of the pro-angiogenic mediator, vascular endothelial growth factor (Vegf) expression, to keratinocytes of the neo-epidermis, mainly adjacent to the wound bed, and this observation was reinforced by immunofluorescence (IF) and RNA in situ hybridization approaches. In accordance is the acute silencing of PKM2 expression by RNA interference in HaCaT-dampened VEGF expression upon growth factor stimulation in vitro. Remarkably, VEGF expression was not modified by altering PKM2 stoichiometry. The proangiogenic role of PKM2 in keratinocytes, which is independent of HIF-1α-expression, as opposed to previous observations in transformed cells, is described in this work.

## Materials and methods

### Materials

Aquatex, DNA oligonucleotide, duplex siRNA oligonucleotide for RNA interference (RNAi), 4′,6-diamidino-2-phenyl-indol-dihydrochlorid, 2-(4-amidinophenyl)-6-indolcarbamidin (DAPI), -dihydrochloridas (D9542-5MG), as well as reagents for molecular bacteriology were purchased from Sigma (Sigma Aldrich, Merck, Darmstadt, Germany). Deoxynucleotide triphosphates of PCR grade were obtained from Roche (Roche Diagnostics, Manheim, Germany). Calcium and magnesium free-Dulbecco’s phosphate buffered saline (PBS), Dulbecco’s modified Eagle medium (DMEM) (high glucose), DMEM/F12, OptiMEM, penicillin/streptomycin (5000 U/ml), sodium pyruvate (100 mM), and Trypsin EDTA (0.05%) were purchased from Gibco (Gibco, Thermo Fisher Scientific Darmstadt, Germany). Fetal calf serum (FCS), was from Biochrom (Biochrom KG, Berlin, Germany). Growth factors and cytokines were obtained from Peprotech (Peprotech, Hamburg, Germany). Lipofectamine 2000 was acquired from Invitrogen (Invitrogen, Thermo Fisher Scientific). Random hexameric primer was obtained from Qiagen (79236; QIAGEN, Hilden Germany), and 2 × qPCRBIO Probe mix Lo-ROX was obtained from Nippon Genetics (NIPPON Genetics Düren, Germany). PKM2 activator thieno-[3,2-b]pyrrole[3,2-*d*]pyridazinone NCGC00186528 (TEPP-46;ML265, PubChem CID 44246499) [28] was obtained from MedChemEexpress (Hoelzel-Biotech, Cologne, Germany).

### Antibodies

Mouse monoclonal antibody (mAb) anti-ß-actin (ACTB) (AC-15) (A5441; Sigma), rabbit anti-Akt (9272; Cell Signaling Technology (CST), Frankfurt am Main, Germany), rabbit mAb anti phospho-Akt (Ser473) (D9E) XP^®^ (4060; CST), rabbit mAb anti-eukaryotic translation initiation factor 4E-binding protein 1 (4E*-*BP1) (53H11) (9644; CST), rabbit anti-phospho-4E*-*BP1 (Ser65) (9451; CST), rabbit anti-eukaryotic initiation factor 2 (eIF2) α (9722; CST), rabbit mAb anti-phosphor-eIF2α (Ser51) (119A11) (3597; CST), rabbit anti-p44/42 MAPK (Erk1/2) (Thr202/Tyr204) (9102; CST), rabbit mAb anti-phospho-p44/42 MAPK (Erk1/2) (Thr202/Tyr204) (20G11) (4376; CST), rat mAb anti-mouse F4/80 (MCA497; BioRad, Munich, Germany), mouse mAb anti-human GAPDH (GT239) (GTX627408; GeneTex, Biozol, Eching, Germany), mouse mAb anti-human glutamine synthase (6/GS) (610517; Becton Dickinson, Heidelberg, Germany), rabbit anti-mouse Hif-1α (NB100-479; Novusbio Biologicals, Wiesbaden, Germany), rabbit anti-human HIF-1α (10006421; Cayman Chemical Biomol, Hamburg, Germany), goat anti-human lamin B (sc-6216; Santa Cruz Biotechnology, Heidelberg, Germany), rat mAb anti-mouse Ly-6B.2 (7/4) (MCA771; BioRad), rat mAb anti mouse Ly-6G (1A8) (BE0075-1; Bio X Cell, Lebanon, NH, USA), rabbit mAb anti PKM1 (D30G6) XP^®^ (7067; CST), rabbit mAb anti-PKM2 (D78A4) XP^®^ (4053; CST), mouse mAb anti-PKM2 (1C11C7) (CoraLite (CL) 488–60268; Proteintech^®^, Chicago, USA), rabbit anti-phosphoPKM (Y105) (3827; CST), rabbit mAb anti-S6 ribosomal protein (5G10) (2217; CST), rabbit mAb anti-phospho-S6 ribosomal protein (Ser235/236) (2F9) (4856; CST), mouse mAb anti-Stat3 (124H6) (9139; CST), rabbit mAb anti-phospho-Stat3 (Tyr705) (D3A7) (9145; CST), goat anti-VEGF (sc-1836; Santa Cruz).

Alexa Fluor™ (AF) 594 conjugated donkey anti-goat IgG (H + L) (A-11058; Invitrogen, Thermo Fisher Scientific).

### Animals

Female C57Bl/6 J (wild-type) mice were obtained from Charles River Laboratories (Charles River Laboratories, Sulzfeld, Germany). At the age of 12 weeks, the mice were caged individually, monitored for body weight, and wounded as described in the following subsection.

### Wounding of mice

Wounding of mice was performed as described previously [29, 30]. Briefly, the mice were anesthetized. Subsequently, six full-thickness wounds (5 mm in diameter, 5 to 7 mm apart) were made on the backs of the mice by excising the skin and the underlying panniculus carnosus. The wounds were allowed to form a scab. An area of 7 to 8 mm in diameter, including the granulation tissue and complete epithelial margins, was excised at the indicated time points for analysis (Fig. S1A). When indicated, wounds were divided into the “wound edge” (“wound margin”), which harbors the hyper proliferative epithelia and the adjacent non-injured skin, and the “wound bed” (“inner wound”), which entails the developing granulation tissue, which consists mainly fibroblasts, polymorphonuclear granulocytes (PMN), monocytes-macrophages, and endothelial cells. The excised skins served as a control. Twelve wounds isolated from four animals were used for RNA analysis. For immunoblot analysis, eight wounds from four individual mice were used. All the animal experiments were performed according to the guidelines and approval of the local ethics animal review board (Regierungspräsidium Hessen, Darmstadt, Germany).

### Cell culture

Cells were cultured in a humidified 5% CO_2_ environment at 37 ℃. Immortalized human keratinocyte HaCaT cell lines [31] were grown in complete DMEM (containing 10% (v/v) FCS and 1% (v/v) penicillin/streptomycin) to confluency before further passaging. Prior to stimulation, cells were brought to quiescence by serum withdrawal for 16–18 h. Human normal epidermal keratinocytes (NHEK) were isolated as previously described [32] and cultured in keratinocyte growth medium 2 (PromoCell, Heidelberg, Germany).

### RNA isolation and real-time PCR analysis of gene expression

RNA isolation was carried out as described previously [30, 33]. Real-time PCR (RT-PCR) was performed using 2 × qPCRBIO Probe mix Lo-ROX (Nippon Genetics). The following pre-designed RT-PCR assays harboring the 6-carboxyfluorescein [Fam] fluorescent dye, and the 6-carboxytetramethylrhodamine quencher were purchased at Applied Biosystems (Thermo Fisher Scientific): Mm01257297_m1 (for mouse Gls), Mm00725701_s1 (for mouse Glul1), Mm00656735-g1 (for mouse G6PDx), Mm00443385_m1 (for mouse HKII), Mm00504650_m1 (for mouse Pfkfb3), Mm00437404_m1 (for mouse Vegf), Hs00173626_m1 (for human VEGF), and HS01000485g1 (for human SOCS3). RT-PCR was performed on ABI Prism 7500 Fast Sequence Detector (Applied Biosystems) as follows: 95 ℃ (2 min); 40 cycles: 95 ℃ (5 s) and 62 ℃ (30 s). Mouse Gapdh quencher (4352339E, VIC) has been use to assess the integrity of the samples. To assess the expression of mouse Pfkfb3, Pgam1, Vdac1, Glud1, and Gapdh as well as the two mouse and human Pkms isoforms by the intercalating fluorescent dye SYBR Green, 5 pmol of the respective forward and reverse primers (see Table 1) and the 2 × SYBR Select mix (4472908; Applied Biosystems) were used. RT-PCR was performed on ABI Prism 7500 as follows: 50 ℃ (2 min) and 95 ℃ (2 min); 40 cycles: 95 ℃ (15 s), 55 ℃ (30 s), and 72 ℃ (60 s). A dissociation stage, 95 ℃ (15 s), 60 ℃ (60 s) 95 ℃ (15 s), and 60 ℃ (15 s), has been added. The specificity of the PCR amplicons was confirmed by melting curve analysis for each amplification product. Analyses of RT-PCR runs were performed by the Sequence Detector software. Relative changes in the respective mRNA expression were normalized to human RPLPO (large ribosomal protein) (4310879E, VIC, human cDNA) and quantified by the 2^−ΔCt^ or 2^−ΔΔCt^ method.

**Table 1:** Oligonucleotide sequence for real-time (RT)-PCR (SYBR Green), cloning, sequencing, and splicing assay, related to material and methods

Oligonucleotides used for RT-PCR (SYBR Green)	
Mus musculus (mm)GAPDH	Fw: 5′ GTGTGAACGGATTTGGCCGT 3′
	Rev: 5′ GAGTGGAGTCATACTGGAACA 3′
mm PKM1 ^a^	Fw: 5′ TTGTGCGAGCCTCCAGTC 3′
	Rev: 5′ ACTCCGTGAGAACTATCAAAGC 3′
mm PKM2 ^a^	Fw: 5′ TTGCAGCTATTCGAGGAACTCCG 3′
	Rev: 5′ CACGATAATGGCCCCACTGC 3′
Homo sapiens (hs)PKM1	Fw: 5′ TGAGGCAGCCATGTTCCAC 3′
	Rev: 5′ ACTCCGTCAGAACTATCAAAGC 3′
hsPKM2	Fw: 5′ TTGCAATTATTTGAGGAACTCCG 3′
	Rev: 5′ GACGATTATGGCCCCACTGC 3′
mm Pgam1	Fw: 5′ GCACTGCCCTTCTGGAATGA 3′
	Rev: 5’ CCTCTTCTGAGAGACCCTCCA 3′
mm VDAC1	Fw: 5’ GGAAGACCAGCTTGCTCGT 3′
	Rev: 5’ CCAGCGATGTCAAAGTCCA 3′
mm Glud1	Fw: 5’ ATCATCAAGCCCTGCAACCA 3′
	Rev: 5’ GCTGTAACGGATACCTCCCT 3′
siPKM2 sequence	
si156 ^b^	Sense [5′ CCAUAAUCGUCCUCACCAA 3’]RNA[TT]DNA
	Antisense [5′ UUGGUGAGGACGAUUAUGG 3′]RNA

^a^After Luo et al. (2011), Cell 145:732-744 [36]

^b^After Goldberg and Sharp (2012), J. Exp. Med. 209:217–227 [34]

### Preparation of whole protein lysates

For whole protein lysate preparation, tissue biopsies and cultured cells were homogenized in a Triton X-100-lysis buffer (1% (v/v) Triton X-100, 20 mM Tris/HCl (pH 8.0), 137 mM NaCl, 10% (v/v) glycerol, 1 mM dithiothreitol, 5 mM EDTA, 10 mM sodium fluoride, 2 mM sodium orthovanadate, 1 mM phenylmethylsulfonyl fluoride, 5 ng/ml aprotinin, 5 ng/ml leupeptin, and 50 nM okadaic acid). Extracts were cleared by centrifugation, and protein concentrations were determined using the BCA Protein Assay Kit (Pierce, Thermo Fisher Scientific).

### Silencing of PKM2 expression by siRNA

HaCaT keratinocytes were cultured in 12-well plates (3E5 cells per well) and grown for 18 h to reach 40–60% confluency. Cells were subsequently transfected with si156 [34] (15 nM) using Lipofectamine 2000 and OptiMEM as described by the manufacturer (see Table 1).

### Pharmacological activation of PKM2

HaCaT keratinocytes were cultured in 6-well plates (5.6E5 cells per well) and grown to confluency. Cells were brought to quiescence by a serum deprived medium as described above. One hour prior to stimulation, the quiescence medium was replaced with a medium containing 50 µM or 100 µM TEPP-46. A medium containing 0.2% DMSO (vehicle) has been used as a control.

### Western blot analysis

Twenty-five to fifty micrograms of protein lysates were analyzed by SDS polyacrylamide gel electrophoresis (PAGE). After transfer to a 0.45-µm nitrocellulose membrane (GE Healthcare, Amersham, Thermo Fisher Scientific) and blocking in 10 mM Tris–HCl (pH 8.0), 150 mM NaCl, and 0.05% (v/v) Tween 20 (TBST) containing 2.5% (w/v) skim milk, specific proteins were identified using primary antibodies for 16 to 18 h at 4 ℃. A secondary antibody [donkey anti-goat (sc-2020; Santa Cruz), goat anti-rabbit (1706515; Bio-Rad), or goat anti-mouse (1706516; Bio-Rad)] conjugated to horseradish peroxidase, and a luminol enhancer detection system (32106, Pierce, Thermo Fisher Scientific) were used to visualize the proteins.

### Blue native gel electrophoresis separation of PKM isoforms

Protein lysates (25 µg) in the Triton X-100 lysis buffer were adjusted to a blue native (BN) PAGE solution, and protein complexes were separated according to their mass on a linear 4 to 13% gradient gel for BN-PAGE as previously described [35].

### Enzyme-linked immunosorbent assay (ELISA)

Cell culture supernatants were collected and cleared by brief centrifugation (300 × g, 5 min at 4 ℃). Fifty microliters of culture supernatants was used for analysis. Quantification of secreted VEGF was performed using human VEGFDuoSet ELISA kits (R&D Systems, Wiesbaden, Germany) according to the manufacturer’s instructions.

### Heidenhain’s AZAN trichrome staining

Wound biopsies were isolated and fixed overnight at 4 ℃ in a 4.0% (v/v) formaldehyde solution, pH 7.0 (ROTI^®^ Histofix 4%, Carl Roth, Karlsruhe, Germany) and embedded in paraffin. Three-micron wound sections were rehydrated and stained in 0.1% (w/v) azocarmine G (Sigma) in 1% (v/v) acetic acid for 7 min, washed briefly with 1% (v/v) acetic acid and followed by oxidation in 5% (w/v) phosphotungstic acid (Sigma) for 20 min and then briefly washed in water. The sections were then counterstained in 1.5% (w/v) aniline blue/orange G (Sigma) for 4 min, briefly washed in water, dehydrated, and mounted in Entellan (Merck, Darmstadt, Germany).

### Immunohistochemistry (IHC)

The three-micron waxed wound sections were rehydrated, and targeted epitopes were unmasked by heat-induced epitope retrieval (HIER) at 100 °C for 20 min (see Table 2) and incubated at 4 °C with primary antibodies overnight. Primary antibodies were detected using either biotinylated rabbit anti-rat antibody (E0468; Dako*,* Hamburg, Germany), or the goat anti-rabbit sc-2018 or donkey anti-goat sc-2023 antibody of the avidin–biotin-peroxidase ImMunoCruz ABC staining system (Santa Cruz) (see Table 2). Antigens were stained by 3,3-diaminobenzidine-tetrahydrochloride (Sigma Aldrich) or by streptavidin–alkaline phosphatase (D0396; Dako) via Permanent AP Red, ZUC001-125 (Zytomed, Berlin, Germany) as chromogenic substrates. Finally, the sections were counterstained with hematoxylin (Mayer’s hematoxylin, Applichem. 254,766.1611 (Applichem, Darmstadt, Germany) and mounted in an aqueous mounting medium, Aquatex. For PKM2 immunostaining, rehydrated tissue sections were washed in IHC-Tris buffer saline (300 mM NaCl, 50 mM Tris–HCl pH 7.6) containing 0.1% (v/v) Tween 20 (TBST), and epitopes were unmasked by boiling in 10 mM citrate (pH 6.0) for 20 min. Tissue’s peroxidases were inactivated by 3% (v/v) H_2_O_2_, nonspecific epitopes were blocked using an animal-serum free blocking solution (CTS 15,019), and the sections were incubated with rabbit mAb anti-PKM2 diluted 1:300 in SignalStain^®^ antibody diluent (CST) at 4 °C overnight. PKM2 epitopes were detected with SignalStain^®^ HRP, Rabbit detection reagents (CST), and stained with SignalStain^®^ DAB substrate (CST). The stained sections were counterstained as above or processed for double immunostaining. For Hif-1αIHC staining, tissue sections were blocked using a biotin/avidin block solution (X0590; Dako) before incubation with a primary antibody and treated by a Dakosignal amplification solution (K1500; Dako) prior to staining by DAB.

**Table 2:** Antibodies used for immunohistochemistry

Antigen	Epitope retrieval procedure	Primary antibody	Biotinylated secondary antibody/solution
F4/80	HIER, 10 mM citrate (pH 6.0)	1:200	Rabbit anti-rat 1:200/DCS^b^
HIF-1α Cayman 10006421	HIER, Dako 10 mM citrate (pH 6.0)	1:10,000	Goat anti-rabbit 1:1000 Vector BA-100/TBST
Ly-6B.2	HIER, 10 mM citrate (pH 6.0)	1:400	Rabbit anti-rat 1:200/DCS
Ly-G	HIER, 10 mM citrate (pH 6.0)	1:150	Rabbit anti-rat 1:200/DCS
PKM2	HIER, 10 mM citrate (pH 6.0)	1:300	CST 8114
VEGF	HIER, Dako TRS ^a^ S1699	1:100	Donkey anti-goat 1:200 ABC

^a^Target retrieval solution; DCS

^b^antibody diluent solution (AL120R; DCS, Hamburg, Germany)

### Immunofluorescence (IF)

Three-micron tissue sections of day 5 wound biopsies were unmasked by HIER using a Dako-target retrieval solution (S1699; Dako), as described above, and incubated with CL 488 labeled mouse mAb anti PKM2 (1:100) and goat anti VEGF (1:100) at 4 °C overnight. Goat anti VEGF antibodies were detected using AF 594 conjugated donkey anti-goat (1:200). Nuclei were stained with DAPI (Sigma) at 1 µg/ml for 60 s, and sections were mounted in a Dako fluorescent mounting medium (S3023; Dako).

### RNA Scope in situ hybridization

RNAScope in situ hybridization on paraffin sections of day 5 wound biopsies was performed using a BaseScope™ duplex detection reagent kit (323810, ACD, Bio-Techne, Minneapolis, MN, USA) and RNAScope H_2_O_2_ and protease reagents (322381, ACD). Tissue sections were unmasked by heat, succeeded by protease IV treatment, each for 15 min exactly according to the manufacturer’s instructions (document number 323800-USM, ACD). The BA-Mm- Vegfa-E2E3 (706421, ACD) and BA-Mm-Pkm-tv2-CE9E10 (714,131-C2, ACD) probes were used to codetect the mRNA expression of *Vegf* and *Pkm2*, respectively, in the wound sections.

### Assessment of PKM2-expressing Ly6B.2 and F4/80 cells in wound sections

Sections of day 3 or day 5 wound biopsies co-stained against activated PMN and monocytes (Ly6B.2) or macrophages (F4/80) and PKM2 were ascertained in four distinct regions of wound edge and wound bed. The percentages of PKM2 expressing cells out of Ly6B.2 or F4/80 positive cells have been assessed.

### Statistical analysis

Data are shown as mean ± SD or ± SEM as indicated. To compare two conditions, unpaired Student’s *t*-test was used for normally distributed homoscedastic data. To compare more than two conditions with unpaired samples, the following statistical tests were used: one-way ANOVA with Bonferroni’s (multiple comparison test) or Dunnett’s (comparison of each condition with control) post-hoc tests for normally distributed homoscedastic data and Kruskal–Wallis test with Dunn’s post-hoc test for data without normal distribution. Data were analyzed using GraphPad Prism software version 5 or 8 (GraphPad Software Inc., San Diego, CA). A *p*-value < 0.05 was considered statistically significant. Significances were indicated as the following: * *P* < 0.05, ** *P* < 0.01, and *** *P* < 0.001.

## Results

### Temporal and spatial activation of metabolic flows during cutaneous wound healing

**Fig. 1:**
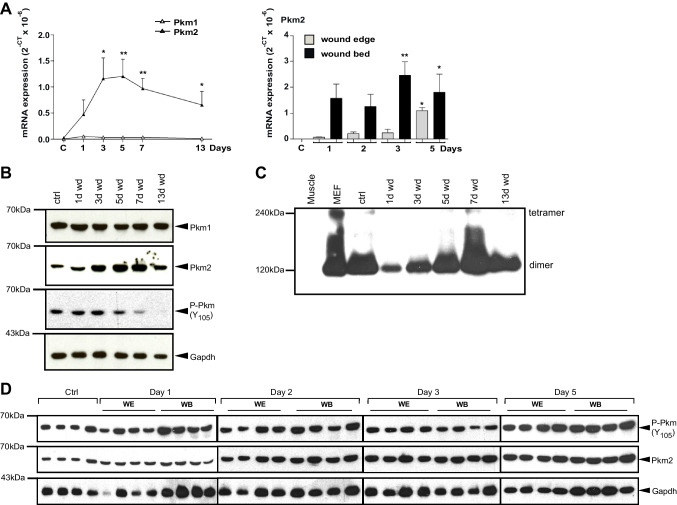
Expression of Pkm isoforms during cutaneous wound healing. **A** Quantification of *Pkm1* and *2* mRNA expression in murine wounds as assessed by RT-PCR (left panel) and *Pkm2* mRNA expression in wound edge (wound margin) and wound bed (inner wound granulation tissue) compartments (right panel) at the indicated time points after injury. Non-wounded skin served as a control (ctrl skin). Bars indicate means ± SD obtained from three wounds (*n* = 3) isolated from four individual animals (*n* = 4). **P < 0.01, *P < 0.05 (ANOVA) as compared to control skin (Dunnett’s post-hoc test). **B** Immunoblot analysis for Pkm isoforms and phosphorylated Pkm(Y_105_) protein expression in murine wounds (wd) at the indicated time points after injury. **C** The stoichiometry of PKM2 as determined by immunoblot analysis of BN-PAGE at the indicated time point. Protein lysates prepared from mouse muscle tissue (Muscle) or mouse embryonal fibroblasts (MEF) were used as a control for Pkm2 expression and stoichiometry. **D** Immunoblot analysis of Pkm2 and phosphorylated Pkm (Y_105_) protein expression in separated wound edge (WE) and wound bed (WB) compartments at the indicated time points after injury. Each individual time point constitutes of wounds (*n* = 8) from individual mice (*n* = 4). The expression of GAPDH was used as a loading control in **B** and **D**. C/ctrl: control skin

The healing of a cutaneous wound is a controlled acute inflammatory process [9]. With minor differences according to the location of the wound (anterior, medial posterior; Fig. S1B and C), the injured skin is nearly perfectly healed within 13 days (Fig. S1A–C). As inflammation and immunity are instructed by metabolic cues [37–39] and inflammatory response is required for proper healing, attention has been drawn in recent years to the metabolic aspects of cutaneous wound repair [39]. Whereas most of our previous works, as well as the majority of other studies dealing with the involvement of various metabolites and/or their respective enzymatic mediators, a mechanistic look at the spatial and temporal roles of metabolic mediator(s) during skin repair is yet inadequately addressed and therefore attracted our attention. We first observed an overall increase in the mRNA expression of enzymes related to glycolysis: Gapdh and Pfkfb3, as well as oxidative phosphorylation: Vdac and the associated hexokinase HkII, which reflected mitochondrial expansion, during the early to the late inflammatory phases i.e., days 3 to 7 after injury (Fig. S2A and B, left panel). Interestingly, the expression of the M2 splice variant of the last glycolytic enzyme, pyruvate kinase, was continuously induced even on day 13 after injury when wound closure is attained, whereas the M1 variant remained unaltered during the whole healing process (Fig. 1A, left panel; Fig. 1B and C). Besides, an increase in glutaminase and glutamate dehydrogenase gene expression reflects an enhanced glutamine anaplerosis of the TCA pathway (Fig. S2C, left panel), which points to arise in anabolic demand of the injured skin. Shunting metabolites toward the pentose phosphate pathway (PPP) seems confined to day 5, as both central genes of this pathway, G6pdx and Pgam1, were found to be specifically expressed at this time point (Fig. S2D, left panel), indicating an increase in oxidative stress during this stage [40]. Markedly, glutamine synthase mRNA and protein were robustly induced immediately after injury and remained at a high level throughout the healing process (Fig. S2E). This fits well with the central role of glutamine metabolism as a carbon source, enabling proliferating cells to utilize glycolysis and TCA intermediates as a source of building blocks for cellular anabolic pathways [41]. Tissue sampling reveals a higher expression of metabolic genes at the wound bed subset than in the wound edge at least until day 5 post wounding, indicating that inflammatory cells are the main source for this pattern of metabolic gene expression. However, 5 days after wounding, an increased expression of these messengers was observed at the wound edge, parallel to the proliferation boost in keratinocytes at the migrating tongue around this time point [9] (Fig. S2A–D, middle and right panels).

### Increased expression of Pkm2 in mononuclear phagocytes and wound keratinocytes

**Fig. 2:**
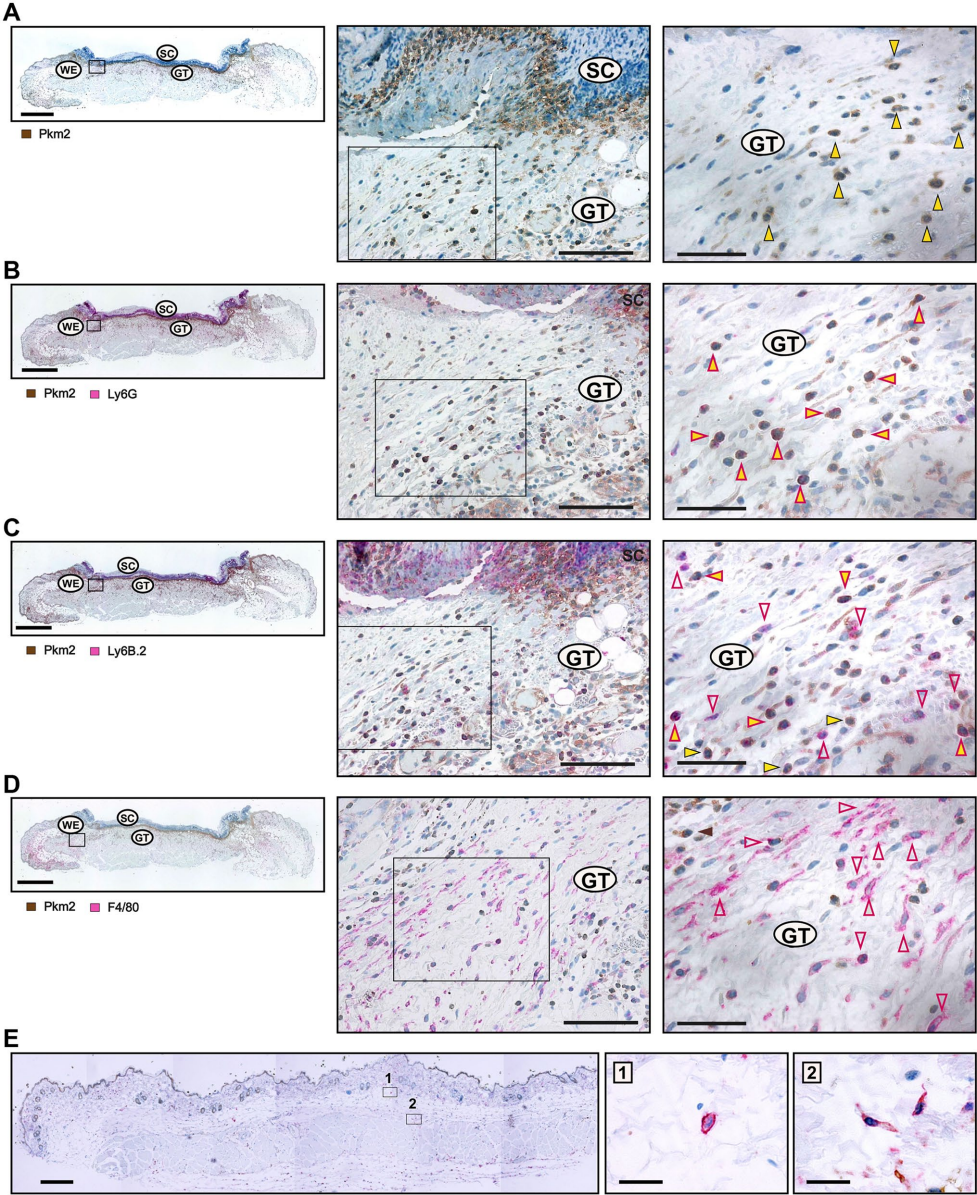
Pkm2 Expression in neutrophils/activated monocytes in early inflammatory wound tissue. Serial sections from day 3 wounds isolated from C57BL/6 J mice using antibodies directed against Pkm2 (brown signals, field yellow arrows) alone (**A**) or in conjugation with antibodies directed against: Ly6G (neutrophils; red signals, red arrows) (**B**), Ly6B.2 (activated PMN/monocytes; red signals, red arrows) (**C**), or F4/80 (macrophage; red signals, red arrow) (**D**). A full wound section is shown at the left side (scale bars: 1000 µm) and the marked region is shown in the middle part (scale bars: 100 µm). A detailed image at higher magnification is shown at the right side (scale bars: 50 µm). WE, wound edge; GT, granulation tissue; SC, scab. A non-injured control-skin, co-stained for Pkm2 and F4/80 is shown in (**E**) (left, scale bar: 500 µm). Higher magnification of resident skin macrophages at [1] and [2] are shown at the right (scale bar: 20 µm)

**Fig. 3:**
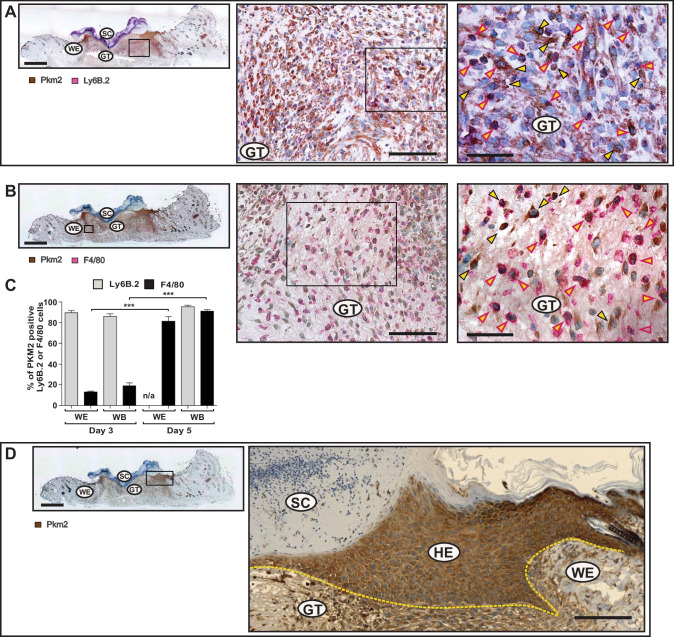
Increasing spread of Pkm2 expression in macrophages and keratinocytes during healing of cutaneous wound. Serial sections from 5-day wounds isolated from C57BL/6 J mice using antibodies directed against Pkm2 (brown signals, field yellow arrows) in conjugation with antibodies directed against: Ly6B.2 (activated PMN/monocytes; red signals, red arrows) (**A**), or in conjugation with antibodies directed against F4/80 (macrophage; red signals, red arrow) (**B**). A full wound section is shown at the left side (scale bars: 1000 µm) and the marked region is shown in the middle part (scale bars: 100 µm). A detailed image at higher magnification is shown at the right side (scale bars: 50 µm). (**C**) Evaluation of Pkm2 expression in neutrophils and mononuclear phagocytes in wound sections obtained from three different mice (*n* = 3) on days 3 and 5 post injury. Ly6B.2 or F4/80 cells were assessed for expressing Pkm2 in 0.72 mm^2^. At least four images taken from wound edge (WE) and two from wound bed (WB) were evaluated for each section. ***P < 0.001; (one-way ANOVA) (Bonferroni’s post-hoc test). Ly6B.2 positive cells are rarely detectable in the wound edge on day 5 post injury and hence not assessed (n/a). Staining of Pkm2 alone is shown in (**D**). A full wound section is shown at the left side (scale bars: 1000 µm) and a detailed image of the marked region is shown at the right side (scale bars: 100 µm). GT, granulation tissue; SC, scab; HE, hyperproliferative epithelia; WE, wound edge; WB, wound bed. The HE is marked by a yellow dash line

The divergent expression of Pkm2 as compared to the Pkm1 splice variant in the wound and its various proposed metabolic and non-metabolic activities [42, 43] prompted us to focus on the function of Pkm2 in cutaneous wound healing. A pro-angiogenic role as a neutrophile secreted factor was indeed suggested for Pkm2 [44]. Yet, our results show an increase and sustained expression of Pkm2 mRNA and protein in later inflammatory stages, when neutrophils decline, whereas proliferation, differentiation, and polarization of keratinocytes, fibroblasts, and macrophages, respectively, take over [45] (Fig. 1B and C). Indeed, in the wound, Pkm2 is mostly expressed as a dimer and hence is metabolically less active (Fig. 1C and Fig. S3A). Furthermore, on day 1 after the injury, stronger phosphorylation on tyrosine 105 (Y105) and hence less activity of Pkm2 have been confined to the wound bed (Fig. 1D). This compartment is mainly populated through migrated neutrophils and platelets at this early inflammatory stage [9]. In this context, it should be noted that Y105 phosphorylation may broaden to the M1 isoform as well (Fig. S3B). Overall, an increasing Pkm2 mRNA and protein expression is detected in the wound edge, which is comprised in part of the hyperproliferative epithelium (Fig. 1A, right panel, and D). Immunohistochemistry staining displays that on day 3 post wounding, leukocytes expressing PKM2 are mainly represented by inflammatory neutrophils and monocytes, which populate the complete wound (Fig. 2A–C). In this early inflammatory phase, wound macrophages scarcely express Pkm2 (Fig. 2D), similar to their counterparts in non-injured tissue, the skin resident macrophages (Fig. 2E). On day 5, inflammatory neutrophils/monocytes are merely at the wound bed (Fig. 3A and C). However, in this mid inflammatory phase, macrophages throughout the whole wound express Pkm2 (Fig. 3B and C). The significance of this transformation of expression pattern is not clear and may reflect a part of polarization process of wound macrophages. Importantly, this staining reinforced that proliferating keratinocytes of the migrating epithelial tongue are a dominant source of PKM2 (Fig. 3D).

### Concomitant expression of Vegf and Pkm2 in wound keratinocytes

**Fig. 4:**
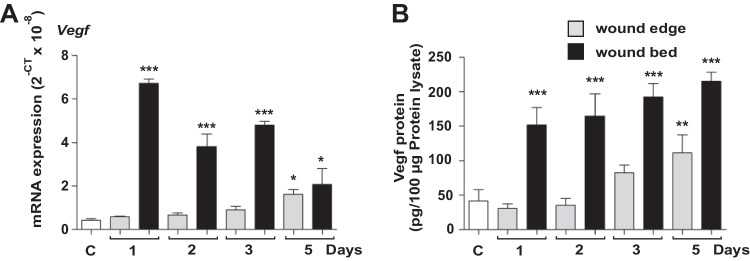
Spatial expression of VEGF during early and mid-wound-inflammation. Quantification of *Vegf* mRNA (**A**) and Vegf_165_ protein (**B**) expression in murine wounds as assessed by RT-PCR (**A**) and ELISA (**B**), respectively, in separated wound edge (WE) and wound bed granulation tissue (WB) compartments at the indicated time points after injury. Non-wounded back skin served as a control (“C”, left-most columns in **A** and **B**). Bars indicate means ± SD obtained from wounds (*n* = 3) isolated from four individual animals (*n* = 4). ***P < 0.001, **P < 0.01, and *P < 0.05 (ANOVA) as compared to control skin (Dunnett’s post-hoc test). C: control skin

**Fig. 5:**
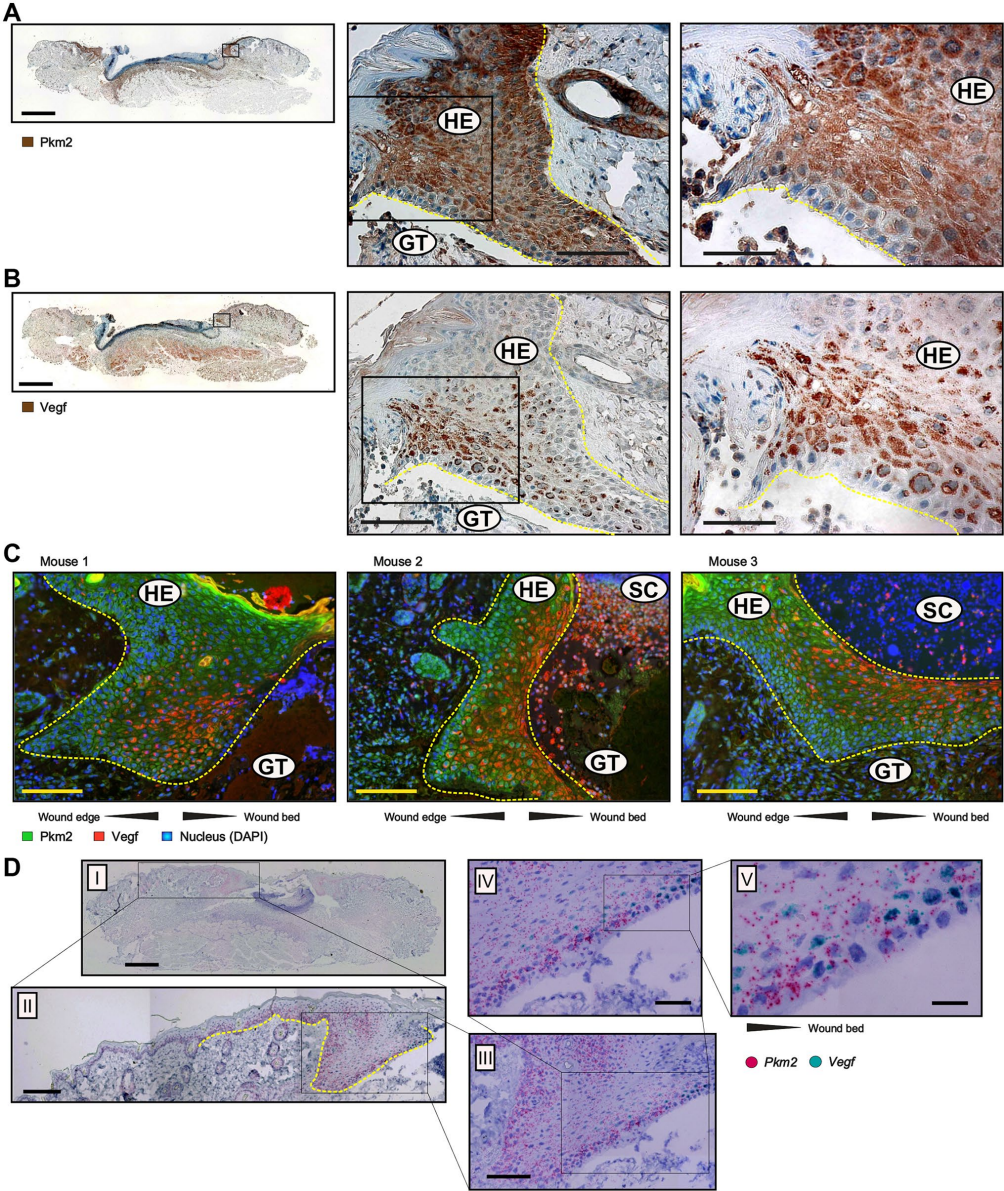
Co-expression of PKM2 and VEGF in keratinocytes of the hyperproliferative epithelial wound tissue. Serial sections from 5-day wounds isolated from C57BL/6 J mice using antibodies directed against Pkm2 **A** or antibodies directed against Vegf **B** (brown signals). A full wound section is shown at the left side (scale bars: 1000 µm) and the marked region is shown in the middle part (scale bars: 100 µm). A detailed image at higher magnification is shown at the right side (scale bars: 50 µm). **C** Immunofluorescence (IF) co-staining of Pkm2 (CL 488, green) and Vegf (AF594, red) in wound sections of day 5 taken from three different mice (scale bars: 100 µm). Nuclei were assessed by DAPI staining (blue). The hyperproliferative epithelium (HE) is marked by yellow dash line. Note that Vegf staining is confined toward wound bed in all three epithelia. GT, granulation tissue; SC, scab. **D** RNA in situ hybridization of *Pkm2* (red dots) and *Vegf* (turquoise dots) mRNA on day 5 wound. Complete wound is shown in I (scale bar: 1000 µm). Enlargement of the left part of the wound is shown in II (scale bar: 200 µm). Expansion of the left hyperproliferative epithelium is shown in III (scale bar: 120 µm). The innermost part of the hyperproliferative epithelium is depicted in IV (scale bar: 60 µm) and a detailed image at higher magnification is shown in V (scale bare: 20 µm)

The expression of Vegf mRNA and protein was temporally and spatially congruent with Pkm2 expression in both wound edge and wound bed sites (Fig. 1A, right panel, Fig. 4A and B, and Fig. S4). During mid-inflammation (mainly on day 5), macrophages in the wound bed are a key source for Vegf in this wound compartment [45]. Of note, on day 5, Vegf expression is significantly increased in the wound edge (Fig. 4). We have previously described keratinocytes of the hyperproliferative epithelium as a major source of VEGF in wound tissue [32]. Immunostaining of serial sections from a wound on day 5 after injury shows Vegf expression co-localization with Pkm2-positive keratinocytes on the wound edge, albeit adjacent to the front of the migrating epithelial tongue (Fig. 5B–D). Similar results were obtained already on day 3 post wounding (not shown). Noteworthy, this expression pattern of Vegf in wound keratinocytes is reminiscent of the suppressor of cytokine signaling (Socs)3 expression in this compartment [46]. We further applied immunofluorescence (IF) staining to gain more insight on how Vegf production is interconnected to Pkm2 expression in keratinocytes of the proliferative epithelium. We used a CoraLite 488-conjugated PKM2-specific mouse monoclonal antibody in combination with an Alexa Fluor 594-conjugate donkey anti goat antibody to co-stain Pkm2 and Vegf, respectively. Co-IF staining of day 5 wound sections from three different mice confirmed the enhanced expression of Pkm2 in the proliferative epithelium and reveals the consistent expression of Vegf in this compartment, mainly in keratinocytes lying toward the wound bed (Fig. 5C). Of note, the patchy expression of the proangiogenic transcription factor Hif-1α is mostly detected in keratinocytes lying adjacent to the wound edge (Fig. S6A), as has been shown, also by Gilles Ponzio and co-workers [47]. Finally, mRNA in situ hybridization using BaseScope™ duplex technology further strengthened this observation and validated the continuously increased expression of *Pkm2* along the rolled proliferative tongue together with Vegf mRNA expression in the keratinocytes sidewise to the wound bed (Fig. 5D).

### The requirement of PKM2 in keratinocytes for growth factor activation of VEGF expression

**Fig. 6:**
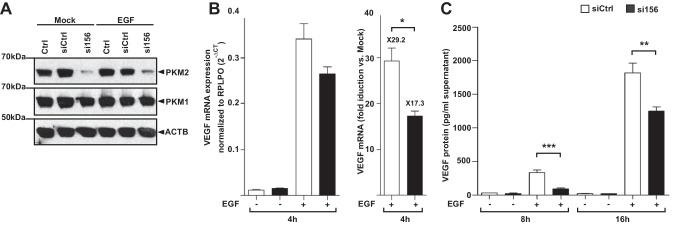
Silencing of PKM2 impedes EGF-induced VEGF expression in HaCaT keratinocytes. **A** HaCaT keratinocytes were transfected with siRNA targeting exon 10 (si156) or control siRNA (siCtrl), and harvested after 48 h; 25 µg of cell lysates were analyzed by immunoblot for the expression of PKM isoforms. Whereas PKM2 protein expression is markedly reduced in si156 transfected cells (upper blot), the expression of PKM1 remained unchanged (middle blot). The expression of ß-actin was used as a loading control. Expression levels of both PKM-isoforms are not affected by EGF (30 ng/ml) stimulation. **B** HaCaT keratinocytes transfected with siRNA against PKM2 (si156) or control siRNA (siCtrl) were treated with EGF (30 ng/ml) for 4 h, and VEGF mRNA was quantified as compared to RPLPO housekeeping gene by RT-PCR (left). EGF stimulation of VEGF mRNA expression in HaCaT keratinocytes is decreased upon silencing of PKM2 as compared to stimulation of control siRNA transfected cells (right). Bars indicate means ± SD obtained from three independent experiments (*n* = 3) performed in triplicate; *P < 0.05 (unpaired Student’s *t*-test). **C** The release of VEGF protein in culture supernatants of HaCaT cells transfected with control siRNA (siCtrl) or siRNA against PKM2 (si156) and stimulated by EGF (30 ng/ml) for the indicated time points was assessed by ELISA. VEGF protein is expressed as pg per ml supernatant. Bars indicate means ± SD obtained from supernatants of three independent cell culture experiments performed in triplicate; ***P < 0.001 and **P < 0.01 (unpaired Student’s *t*-test)

Elevated expression of PKM2 in the hyperproliferative epithelium may suggest a functional role for PKM2 driving VEGF expression in keratinocytes. Similar to normal human epidermal keratinocytes (NHEK), HaCaT cells express higher levels of PKM 1 and 2 isoforms, of which PKM2 is more strongly expressed in both strains, and this phenomenon is intensified in HaCaT as emerged from RNA and protein data (Fig. S5A and B). In cells expressing HIF-1α under hypoxic conditions or due to malfunctioning HIF-1α degradation signal, as in von-Hippel-Lindau (VHL) null renal carcinoma cells (RCC4), PKM2 has been shown to regulate the HIF-1α metabolic and angiogenic settings via enhancing HIF-1α binding to hypoxia response elements (HREs) within metabolic regulatory genes such as *LDHA* and *PDK1* as well as *VEGF* [36]. However, the expression of Hif-1α is uneven in keratinocytes of the hyperproliferative epithelium tongue (Fig. S6A) [47], implying an additional mode to accelerate VEGF expression in this compartment, apart from HIF transcriptional program. Along this line, the expression of HIF-1α in HaCaT cells is restricted to hypoxia or hypoxia mimicry by CoCl_2_ [48] (Fig. S6B). To ascertain the contribution of PKM2 in keratinocyte-driven angiogenic program, we have performed a knockdown of PKM2 using small interfering (si) RNA designed to target PKM2 specifically [34] without affecting PKM1 levels (Fig. 6A), and this approach has been employed to study the role of PKM2 in VEGF expression by HaCaT keratinocytes. To this end, we stimulated HaCaT cells with epidermal growth factor (EGF), a key effector of epithelial cells during wound repair [49] and a potent activator of VEGF_165_ splice variant (hereafter VEGF) expression and secretion in cultured keratinocytes [50, 51]. Partial knockdown of PKM2 dampened EGF to induce VEGF mRNA (Fig. 6B), and this effect was more pronounced at the VEGF protein level (Fig. 6C). Increased PKM2 dimer and tetramer formation by PKM2 activator TEPP-46 has no detectable effect on VEGF expression (Fig. S7), indicating the importance of the total PKM protein rather than their overall stoichiometry for maximal induction of VEGF in HaCaT keratinocytes.

### Silencing of PKM2 partially alters EGF signal transduction in keratinocytes

**Fig. 7:**
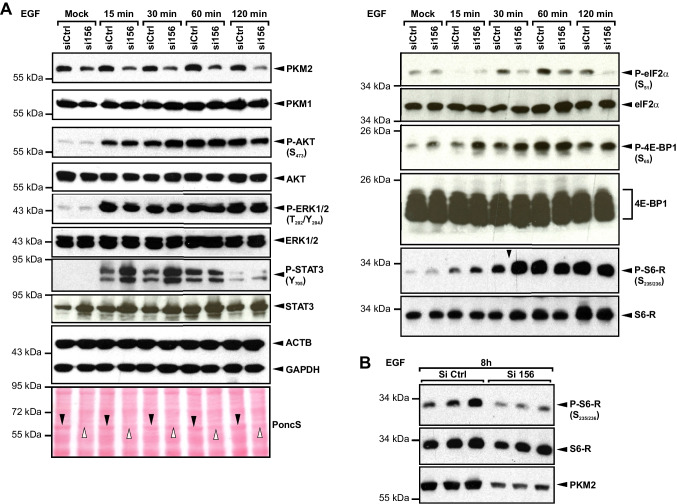
Silencing of PKM2 dysregulates growth factor activation of STAT3 and translational regulators. **A** HaCaT keratinocytes were transfected with PKM2-specific siRNA (si156) or control siRNA (siCtrl), and stimulated after 48 h by 30 ng/ml EGF for the indicated time points, and 25 µg of cell lysates were analyzed by immunoblot to assess the phosphorylation state of PKB (AKT) (Ser_473_), ERK1/2 (Thr_202_/Tyr_204_), STAT3 (Tyr_705_), eIF2α (Ser_51_), 4E*-*BP1 (Ser_65_), and S6 ribosomal protein (S6RP) (Ser_235/236_), and the expression level of the corresponding total protein. The silencing of PKM2 expression was verified by immunostaining of PKM2 and PKM1 proteins (top and second blots, respectively, left panel). The expression of ß-actin and GAPDH was used as a loading control. Ponceau S staining (PoncS, left panel, last blot) shows a decrease of a 60-kDa band (black downward arrowheads), subsequent to transfection with si156 (white upward arrowheads). A representative blot from three independent experiments is shown. **B** The phosphorylation state of S6RP (Ser_235/236_) at 8 h of stimulation by 30 ng/ml EGF was assessed in 25-µg cell lysate. Staining of total S6RP has been used as a loading control. PKM2 expression is shown in the lower blot. Results from three (*n* = 3) independent experiments are presented

As pointed out, PKM2 has been shown to act through modulation of the transcriptional program to regulate glucose metabolism, Warburg effect, growth, and survival [52, 53]. In our setting, transient reduction of PKM2 expression in keratinocytes hampers full induction of transcription and, to a greater extent, translation of VEGF by EGF. Therefore we examined the effect of PKM2 attenuation on the activation of key signal transducers that were shown to regulate VEGF production in keratinocytes, following growth factor stimulation in vitro and during wound healing in vivo [54]. The silencing of PKM2 neither affected AKT nor ERK activation; however, it increased STAT3 phosphorylation and, to a minor degree, its expression (Fig. 7A, left panel). Unexpectedly, the eukaryotic initiation factor 2 (eIF2)α, which stimulates the translation of capped mRNA (e.g. VEGF), was found to be hypophosphorylated and hence activated. On the other hand, the repressor of cap-dependent, 4E-BP1 was slightly more phosphorylated and therefore inhibited upon knockdown of PKM2 (Fig. 7A, right panel). Impressively, the activation of the initiation of translation machinery-accompanied intense STAT3 phosphorylation was much stronger upon stimulation of PKM2 knockdown cells with bona fide wound healing cytokines IL-6 and IL-22 (Fig. S8). The higher activation of the translational machinery could represent a compensatory response in PKM2 knockdown cells. Recently, activation of AKT has been proposed to protect cancer cells from growth inhibition induced by PKM2 knockdown [55]. As indicated here, AKT activation remained unchanged upon PKM2 silencing but additional compensation mechanisms cannot be ruled out. Even more surprising was the earlier phosphorylation of S6 ribosomal protein (S6RP) upon EGF stimulation in cells with knockdown of PKM2, which peaked already at 30 min (Fig. 7A, right panel, penultimate blot). However, this activation state of S6RP declined more rapidly in PKM2 knockdown cells than in the control cells (Fig. 7A, right panel, penultimate blot, and Fig. 7B). Earlier deactivation of S6RP halts the ribosome in PKM2 knockdown cells and could be accounted for the pronounced reduction of VEGF protein level in these cells. Of note, visualization of the blotted protein lysates of control- and PKM2-siRNA transfected cells using Ponceau S staining revealed the abundance of PKM2 in keratinocytes. The weakening of a 60-kDa protein band, corresponding to PKM2, could be easily seen (Fig. 7A, left panel, last blot and Fig. S8 left and right panels, last blots).

### Silencing of PKM2 augments STAT3 signaling in keratinocytes

**Fig. 8:**
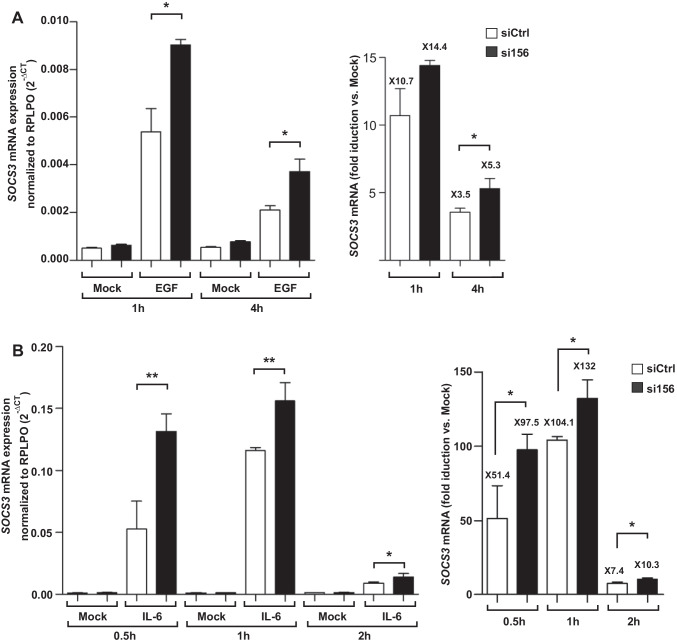
Enhanced activation of SOCS3 mRNA in PKM2 knockdown keratinocytes. **A** The expression of SOCS3 mRNA in EGF-stimulated HaCaTs was assessed by RT-PCR (left), and the fold induction of enhanced expression of SOCS3 in PKM2 knockdown cells as compared to stimulation of control siRNA transfected cells was evaluated by the 2^−ΔΔCt^ method (right). Bars indicate means ± SD obtained from three independent experiments (*n* = 3) performed in triplicate; *P < 0.05 (unpaired Student’s *t*-test). **B** The expression of SOCS3 mRNA upon stimulation with IL-6 (20 ng/ml) was quantified by RT-PCR (left). IL-6 stimulation of SOCS3 mRNA expression is markedly increased upon targeting PKM2 expression as compared to stimulation of control siRNA transfected cells (right). Bars indicate means ± SD obtained from three independent experiments (*n* = 3) performed in triplicate; **P < 0.01 and *P < 0.05 (one-way ANOVA) (Bonferroni’s post-hoc test). The fold of stimulation in **A** and **B** is indicated on top of the bars (**A** and **B**)

The enhanced phosphorylation of STAT3 by growth factor impetus or inflammatory stimuli is a part of an intact activated STAT3 signaling cascade. Nevertheless, the pronounced phosphorylation of STAT3 following stimulation of PKM2-silenced keratinocytes is not explicable. Therefore, to confirm that this amplified phosphorylation is also functionally manifested by increasing STAT3 transcriptional activity, we analyzed the expression of the suppression of cytokine signaling (SOCS)3, a surrogate marker for STAT3 activation. Remarkably, the silencing of PKM2 significantly augmented the activation of *SOCS3* mRNA transcription in response to EGF (Fig. 8A). Stimulation of SOCS3 expression is only secondary to EGF signaling cascade, ensuing STAT3-activation. As a rapid immune modulator, SOCS3 is primarily induced by inflammatory cytokines (via STAT3) in the course of a negative feedback control mechanism. Indeed, aligned with the increased induction of STAT3 phosphorylation by pro-inflammatory cytokines in PKM2 knockdown cells (Fig. S8), silencing of PKM2 sharply enhances IL-6 stimulation of *SOCS3* mRNA expression (Fig. 8B). This raised the induction of *SOCS3*, as opposed to the reduced stimulation of VEGF implying a specific effect of PKM2 to accomplish VEGF production in keratinocytes. Generally, inflammatory stimuli escalate wound angiogenesis [56] and IL-6 amplifies growth factor-stimulation of VEGF expression in keratinocytes (Fig. S9), ruling out an inhibitory effect of SOCS3 and probably other STAT3-regulated gene products on VEGF expression.

## Discussion

Wound healing is a multi-stage process orchestrated by complicated interactions between cellular and humoral inflammatory cues. It involves cell infiltration, proliferation, expulsion, and death as well as destruction of damaged structures and assembly of scaffolds for tissue repair [57]. In a way, wound healing reminisces neoplasia, but resolved wound healing is a well-coordinated process, whereas neoplasia is uncontrolled growth [27]. The altered metabolic landscape of cancer cells as compared to normal tissue has drawn the attention of researchers in the field of tumor biology, since the early studies of Otto Warburg almost a century ago [2, 58]. In analogy to cancer, altered metabolism was shown to affect the healing outcome, and along with this finding, injured tissue and neoplasia have been found to share common nodes [10, 54, 59]. Initially, this work tended to gain an overall idea on the metabolic nature of wound inflammation. Through following the expression profile of central enzymes involved in glycolysis, oxidative phosphorylation, the pentose phosphate shunt, and glutamine anaplerosis, a mixed picture has emerged, suggesting a spatially and temporarily regulated co-activation of metabolic pathways that support various polarization modes and not a particular one as we understand it today in other inflammatory conditions [60]. Notably, the expression of enzymes connected to the pentose phosphate shunt was confined to day 5 after injury, indicating a sharp rise in oxidative stress and the demand to counterbalance it at this stage. Of interest, we analyzed the expression mode of Pkm2, which, as opposed to its constantly expressed alternative splice variant and metabolic active Pkm1, was markedly induced up to day 3 after wounding. Whereas the relatively high phosphorylated state of Pkm2 at an early time point implies low activity, the injured tissue is governed by neutrophils. Its increased expression in macrophages and keratinocytes of the hyperproliferative epithelia, later on during mid-inflammatory phase as detected histologically, indicates an acute role of Pkm2 in inflammation and re-epithelialization of the damaged skin. Accordingly, the dimer stoichiometry of Pkm2 (Fig. 1) implies a reduced pyruvate kinase activity that is connected to intense cell proliferation [4]. The hyperproliferative epithelium was indicated as a source for Vegf to drive angiogenesis during acute healing [32]; hence, the co-localization of Vegf expression in a subset of keratinocytes in this compartment was not unexpected (Fig. 5). However, as opposed to the patchy expression pattern of the pro-angiogenic transcription factor Hif-1α, proximal to the wound edge (Fig. S6A),Vegf is homogenously expressed in keratinocytes proximal to the wound bed, as confirmed by IF and RNA in situ hybridization (Fig. 5C and D). This further supports the notion that the factors released from the wound bed (e.g., cytokines/chemokines) are required to stimulate Vegf expression in keratinocytes of the hyperproliferative epithelium. A strong association between PKM2 and VEGF expression has been proposed by Luo et al. in VHL null renal cell carcinoma a decade ago [36]. This important work provided an exo-metabolic mechanistic explanation that connects PKM2 to the enhancement of HIF-1α-dependent genomic reprogramming, including VEGF expression. However, as noted above, Vegf expression in wound keratinocytes does not necessarily correlate with the expression of Hif-1α (Fig. S6A) [48], and HaCaT keratinocytes do not express HIF-1α unless stimulated by hypoxic or inflammatory signals (Fig. S6B) [61]. In this context, we may recall the research by Vander Heiden and his co-workers who showed that in mouse tumor-derived cell lines, PKM2 was dispensable for hypoxia-induced gene expression [62]. In addition, Luo and colleagues targeted exon 6 to knockdown PKM gene product(s) and hence silenced both PKM1 and 2. Acute silencing of PKM2 in HaCaT by siRNA, specifically targeting exon 10, reveals a reduction in stimulation of VEGF mRNA and, to a greater extent, protein expression upon induction by growth factors. Importantly, the silencing of PKM2 per se leads to defined changes in signaling molecules, which we do not fully understand. Whereas STAT3 and eIF2α were induced, 4E-BP1 was inhibited, and the activation of S6RP was dampened, albeit at a later time point. While the reduction of S6RP activation parallel to PKM2 silencing matches the drop in VEGF-stimulated levels, induction of eIF2α concomitant to suppression of 4E-BP1 contradicts this result. Counting the inducing versus suppressing roles of eIF2α and 4E-BP1, respectively, in the translation of capped mRNAs, it was suggested that the above modification may reflect a cellular stress response, which leads at a later time point to attenuation of S6RP activation.

Still, another ambiguity is the enhancement of STAT3 phosphorylation following growth factor or inflammatory stimuli of PKM2 knockdown cells. The enhanced activation of STAT3 due to acute silencing of PKM2 manifests in higher transcriptional activation of *SOCS3* mRNA by EGF or inflammatory stimuli. However, decreasing of VEGF production due to increased expression of SOCS3 can be ruled out as IL-6 rises the stimulation of VEGF expression by EGF, concomitantly to induction of *SOCS3* mRNA expression (Figs. 8B and S9). STAT3 was among the first proteins that were appeared to be phosphorylated and activated by nuclear PKM2 [63]. Later on, this capability of PKM2 to phosphorylate effector proteins and thereby modulate their activity has been revised [24]. Nevertheless, activation of STAT3 by PKM2has been further suggested in many works. Recently, Alves-Filho and his co-workers showed that Pkm2 contribute to autoimmune inflammation in experimental autoimmune encephalomyelitis (EAE) via fine-tuning Stat3 activation [64]. Pharmacological activation of Pkm2 metabolic action via induction of tetramer formation by the small molecule activator TEPP-46 reduces Pkm2 nuclear translocation and Stat3 phosphorylation, thereby limiting CD4^+^T helper 17 (Th17) cell formation and ameliorating EAE. Similarly, Luke O’Neill and his co-workers provided evidence that tetramerization of Pkm2 by TEPP 46 limits the development of both Th17 and Th1 cells [65] and thereby restrains aggravating inflammation in EAE. However, their finding was independent of Stat3 or Stat1 activation. Similarly, metabolic activation of Pkm2 has been shown to restrain pro-inflammatory activation of murine macrophages by reducing the formation of Hif-1α-Pkm2 ternary complex at IL-1β promoter [66] or induce mitochondrial biogenesis [67]. Interestingly, activation of PKM2 tetramer formation TEPP-46 did not alter VEGF production in EGF-stimulated HaCaT cells (Fig. S7). EGF alone does not alter the stoichiometry of PKM2 (Fig. S7B) and barely induces phosphorylation of tyrosine 105 as the inflammatory cytokine IL-6 does (not shown). It is therefore tempting to assume that in keratinocytes the acute amount of PKM protein determines the full activation of VEGF expression. Curiously, on examining Ponceau S staining blotted HaCaT protein, we were surprised to note the weakening of a 60-kDa band, corresponding to the size of PKM2 of cell lysates, transiently knockdown PKM2 (lower blots in Fig. 7A, right panel, and Fig. S8A and B). Considering this 60-kDa species as the M2 splice variant of pyruvate kinase suggests an unusually high level of PKM2 protein expression in HaCaT keratinocytes. Along this line, in an accompanied study, we have shown that gene editing-mediated inactivation of the splicing of exon 10 leads to abandoned expression of PKM2 and yet does not result in alteration of VEGF induction, in part due to nearly sevenfold concomitant induction of PKM1 expression [68].

In summary, our study proposes a supportive role for PKM2 to facilitate the induction of angiogenesis by growth factors in keratinocytes, which is independent of HIF-1α. Furthermore, acute silencing of PKM2 in keratinocytes causes altered activation of transcriptional and translational factors stimulated by EGF and inflammatory cytokines. This culminated in the reduced stimulation of VEGF expression in keratinocytes.

## Supplementary Information

Below is the link to the electronic supplementary material.

Supplementary file1 (PDF 666 KB)

Supplementary file2 (PDF 250 KB)

Supplementary file3 (PDF 76 KB)

Supplementary file4 (PDF 27 KB)

Supplementary file5 (PDF 59 KB)

Supplementary file6 (PDF 1275 KB)

Supplementary file7 (PDF 95 KB)

Supplementary file8 (PDF 383 KB)

Supplementary file9 (PDF 94 KB)

Supplementary file9 (DOCX 43 KB)

## Data Availability

The data presented in this work are available upon request from the corresponding author.

## Abbreviations

PKM2: Pyruvate kinase 2
OXPHOS: Oxidative phosphorylation
HE: Hyperproliferative epithelium
VEGF: Vascular endothelial growth factor
