# Supplementary figures and images for: Expression of PKM2 in wound keratinocytes is coupled to angiogenesis during skin repair in vivo and in HaCaT keratinocytes in vitro
Source: J Mol Med (Berl). 2023 Jan 12;101(1-2):151–69. doi: 10.1007/s00109-022-02280-6 (PMC9977898; doi:10.1007/s00109-022-02280-6)

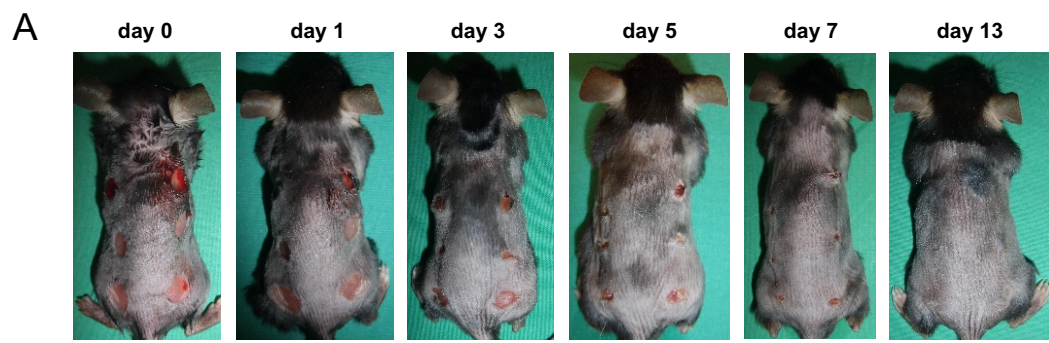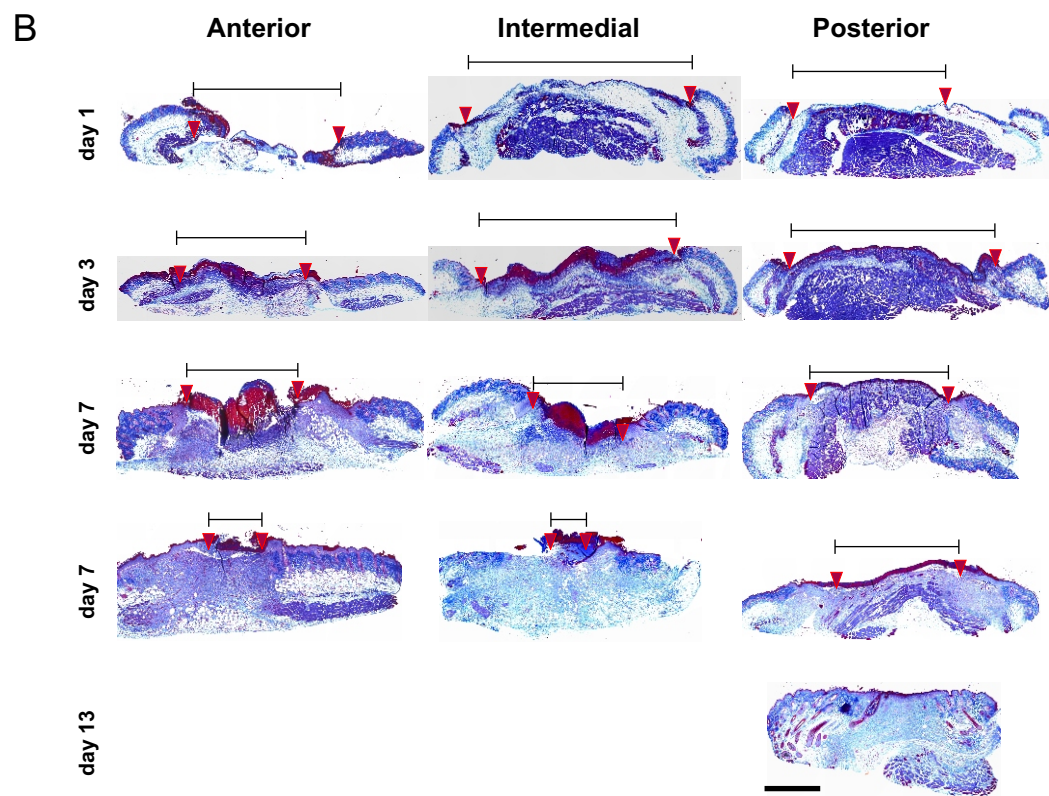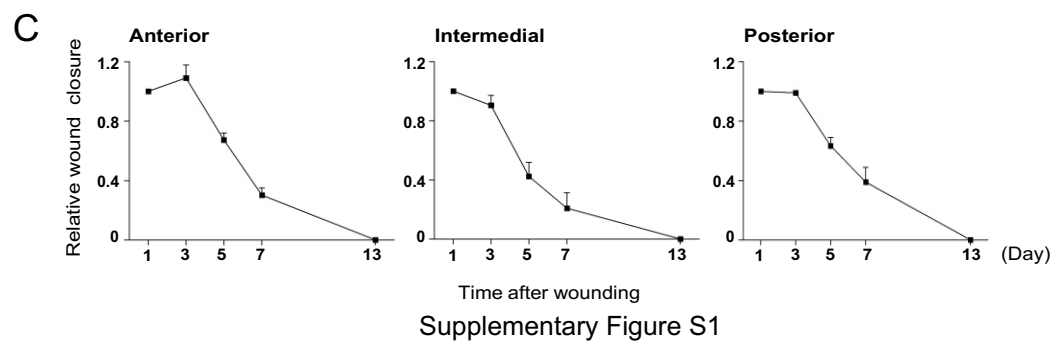

Supplement: Supplementary file 1 — Supplementary file1 (PDF 666 KB) [file 109_2022_2280_MOESM1_ESM.pdf]

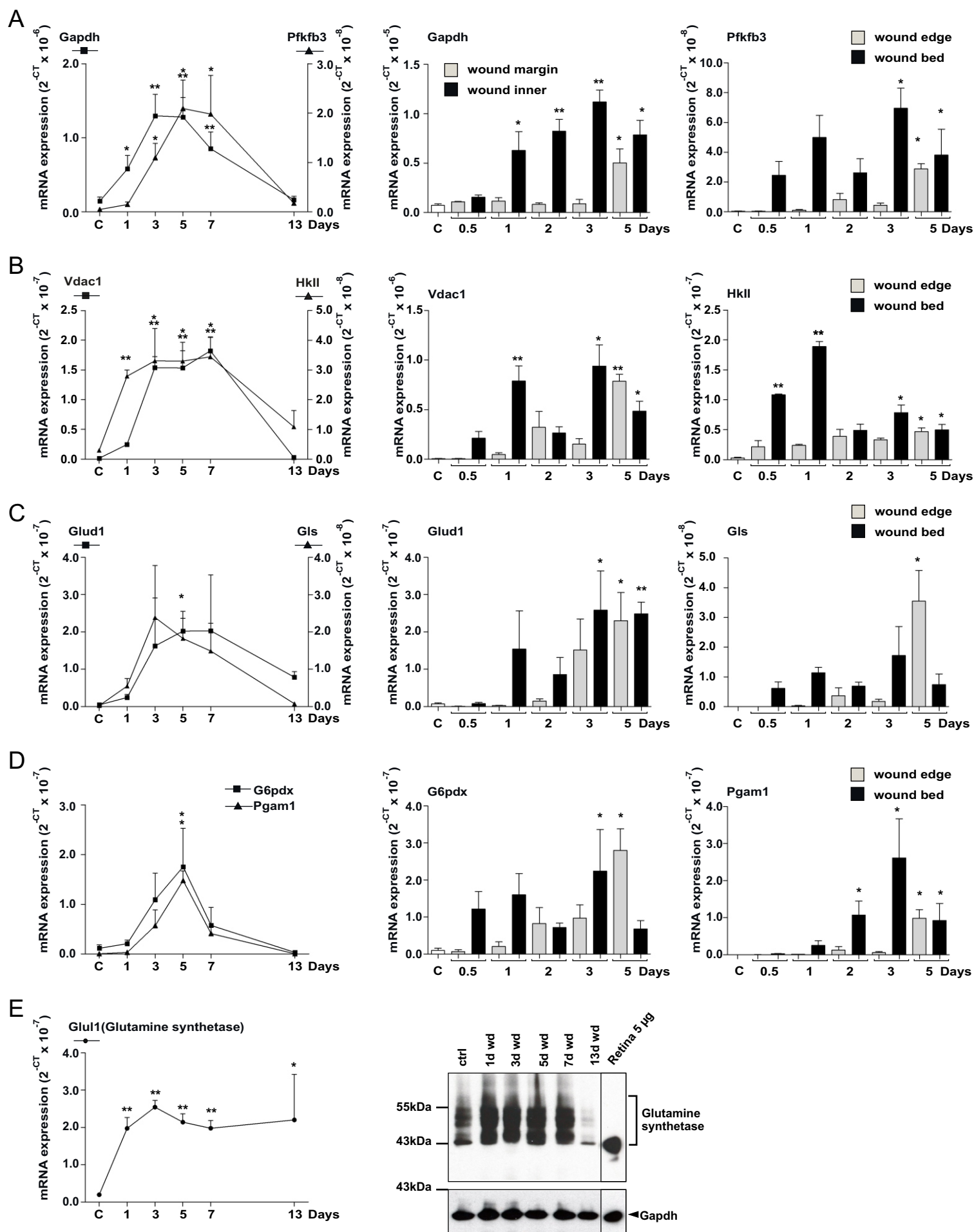

Supplementary Figure S2

Supplement: Supplementary file 2 — Supplementary file2 (PDF 250 KB) [file 109_2022_2280_MOESM2_ESM.pdf]

**A**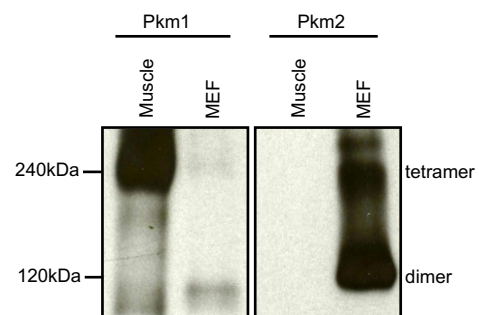**B**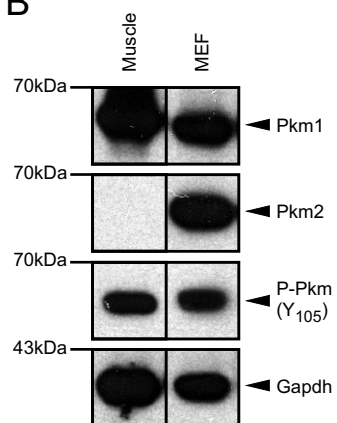

Supplementary Figure S3

Supplement: Supplementary file 3 — Supplementary file3 (PDF 76 KB) [file 109_2022_2280_MOESM3_ESM.pdf]

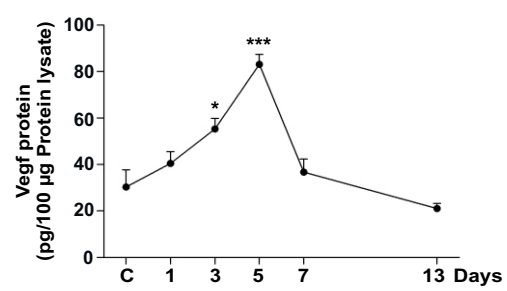

Supplementary Figure S4

Supplement: Supplementary file 4 — Supplementary file4 (PDF 27 KB) [file 109_2022_2280_MOESM4_ESM.pdf]

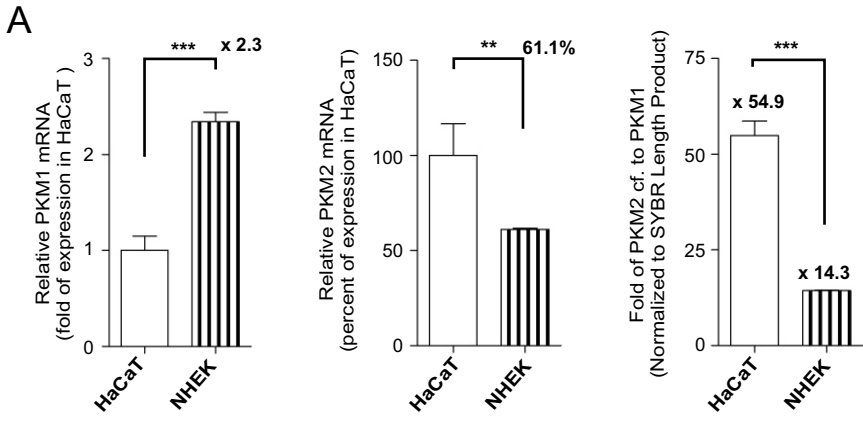

Supplementary Figure S5

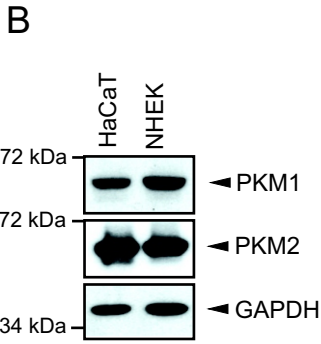

Supplement: Supplementary file 5 — Supplementary file5 (PDF 59 KB) [file 109_2022_2280_MOESM5_ESM.pdf]

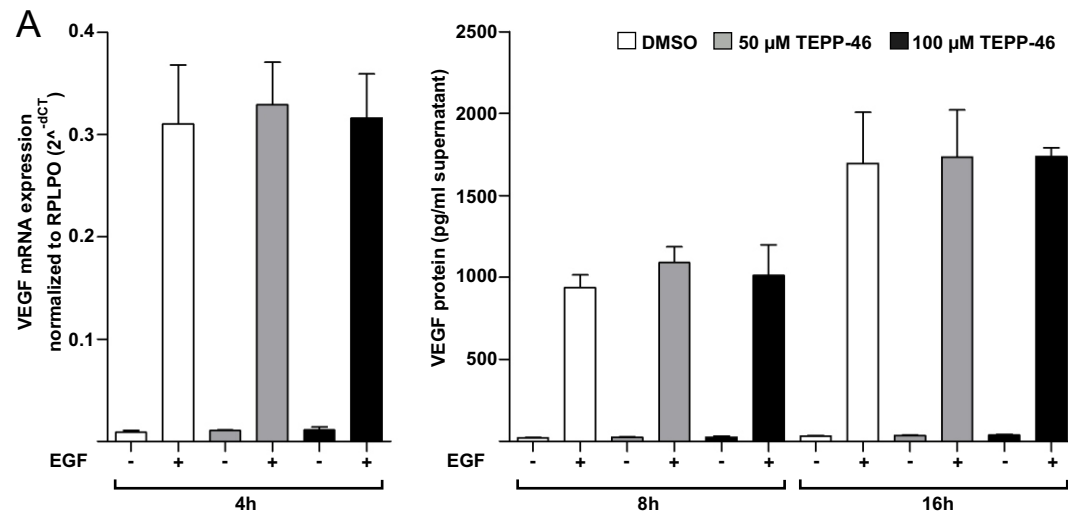

Supplementary Figure S7

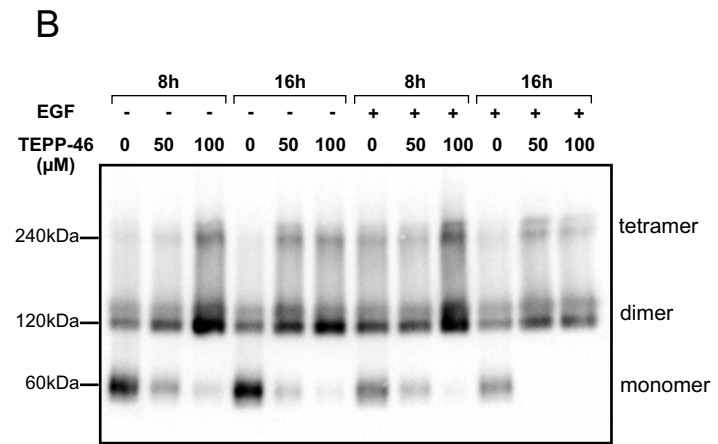

Supplement: Supplementary file 7 — Supplementary file7 (PDF 95 KB) [file 109_2022_2280_MOESM7_ESM.pdf]

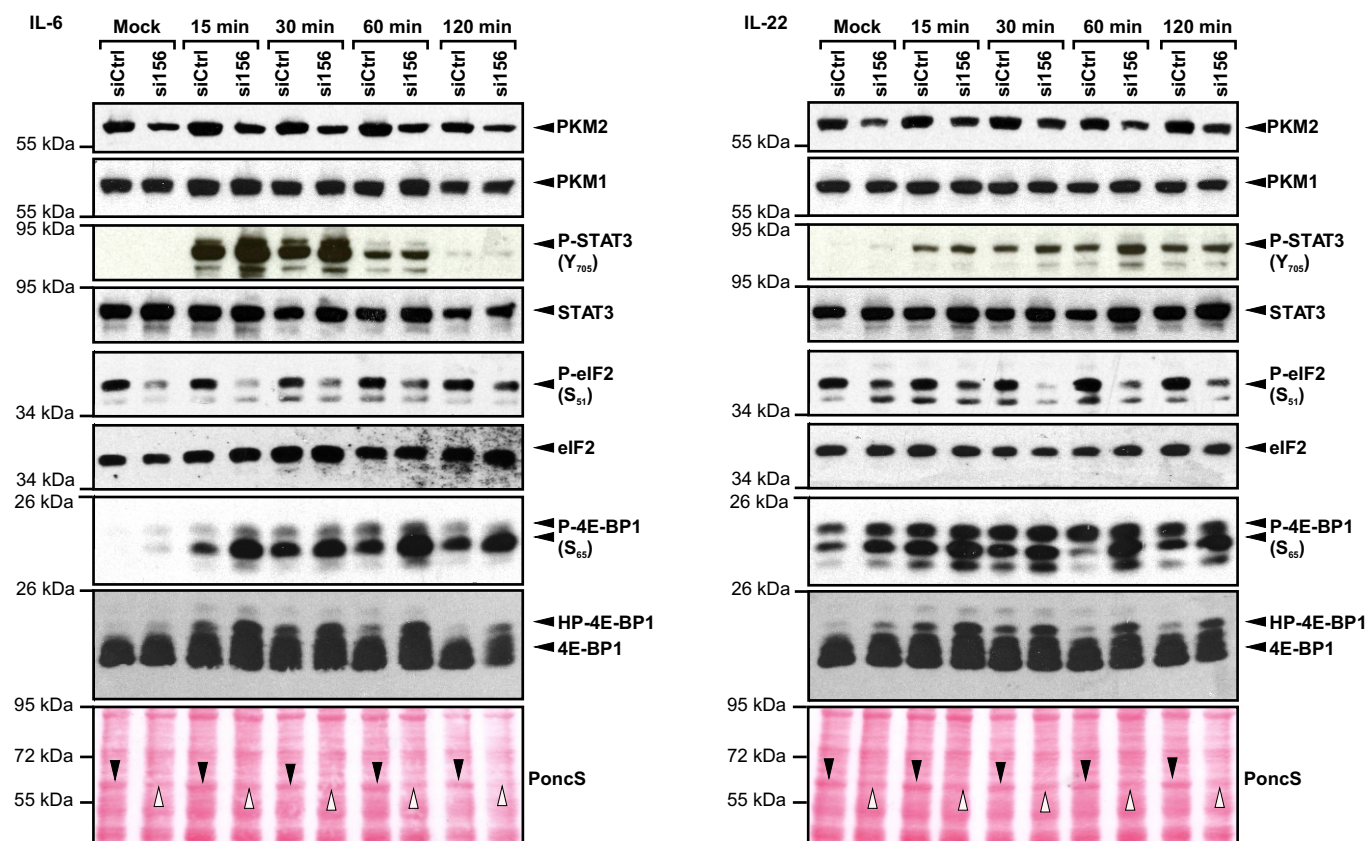

Supplementary Figure S8

Supplement: Supplementary file 8 — Supplementary file8 (PDF 383 KB) [file 109_2022_2280_MOESM8_ESM.pdf]

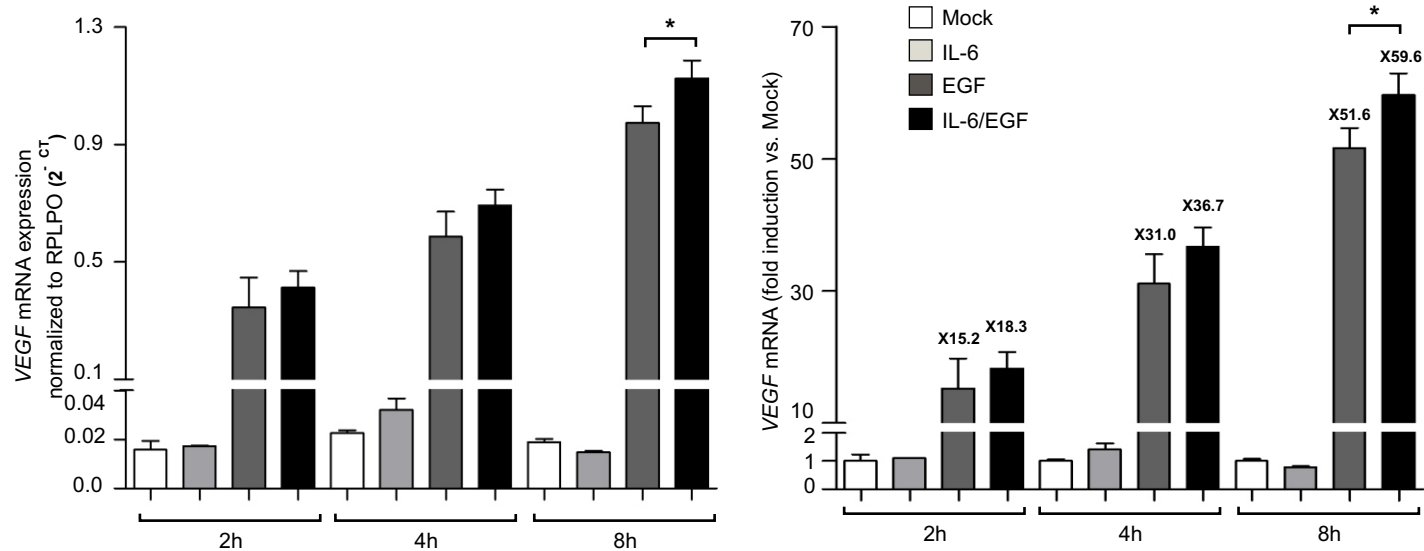

Supplementary Figure S9

Supplement: Supplementary file 9 — Supplementary file9 (PDF 94 KB) [file 109_2022_2280_MOESM9_ESM.pdf]
