# Supplementary material for: Expression of PKM2 in wound keratinocytes is coupled to angiogenesis during skin repair in vivo and in HaCaT keratinocytes in vitro
Source: J Mol Med (Berl). 2023 Jan 12;101(1-2):151–69. doi: 10.1007/s00109-022-02280-6 (PMC9977898; doi:10.1007/s00109-022-02280-6)
Supplement: Supplementary file 6 — Supplementary file6 (PDF 1275 KB) [file 109_2022_2280_MOESM6_ESM.pdf]

A

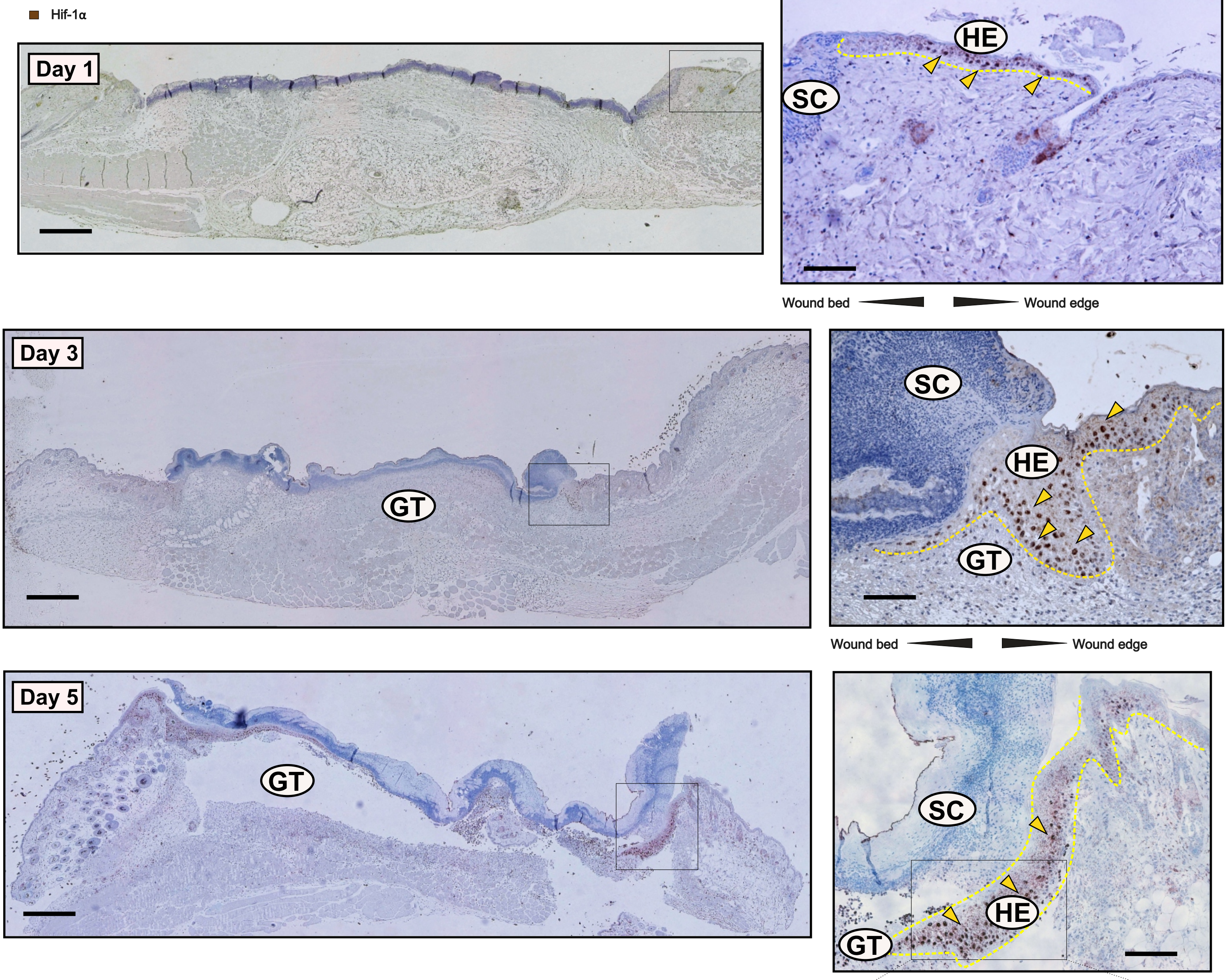

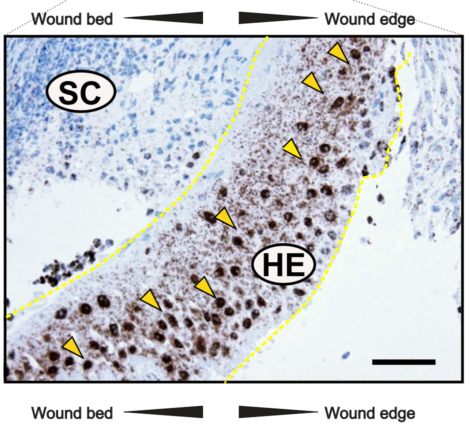

B

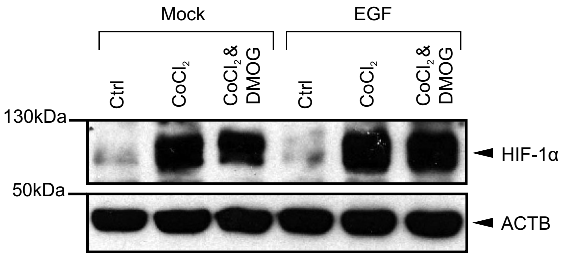

Supplementary Figure S6
