# Supplementary material for: Expression of PKM2 in wound keratinocytes is coupled to angiogenesis during skin repair in vivo and in HaCaT keratinocytes in vitro
Source: J Mol Med (Berl). 2023 Jan 12;101(1-2):151–69. doi: 10.1007/s00109-022-02280-6 (PMC9977898; doi:10.1007/s00109-022-02280-6)
Supplement: Supplementary file 10 — Supplementary file9 (DOCX 43 KB) [file 109_2022_2280_MOESM10_ESM.docx]

**Expression of PKM2 in wound keratinocytes is coupled to angiogenesis during skin repair *in vivo* and in HaCaT keratinocytes *in vitro***

**Supplemental Information**

**Figure S1. Progress of cutaneous wound healing in the mouse.** (A) Recording of healing process in C57BL/6J mice from injury (day 0, left) to complete healing (day13, right). (B) Transverse wound section from day 1 to day 13 at the indicated position, were visualized by Heidenhain's AZAN trichrome staining. The expanse between the proliferative epithelia of the wound edges has been verified as the wound’s diameter (scale bar: 1000 µm). (C) The wound diameter at each time point has been determined and presented as quotient of the average diameter of the wounds at day 1 in the same position. The spatial (i.e. anterior medial and posterior) and temporal rate of wound closure is shown. Bars indicate means ± SEM obtained from wound sections isolated from three (n=3) individual animals.

**Figure S2. Temporal and spatial induction of key metabolic genes during cutaneous wound healing.** Temporal (left) and spatial (right) mRNA expression of the glycolytic enzymes glyceraldehyde-3-phosphate dehydrogenase (Gapdh, left axis) 6-phosphofructo-2-kinase/fructose-2,6-biphosphatase 3 (Pfkfb3, right axis) (A); mitochondrial and mitochondrial associated proteins voltage-dependent anion channel 1 (Vdac1, left axis) and hexokinase II (HKII, right axis) (B); the glutamine anaplerosis enzymes, glutamate dehydrogenase 1 (Glud1, left axis) and glutaminase 1 (Gls1, the “kidney isoform”, right axis) (C); and the pentose phosphate shunt factors, glucose-6-phosphate dehydrogenase (X-linked) (G6pdx) and phosphoglycerate mutase 1 (Pgam1) (D) in murine wounds as assessed by RT-PCR. (E) The mRNA expression of the essential glutamine producing enzyme, glutamine synthase (glutamate ammonia ligase) (Glul1) is shown in the left part. Immunoblot analysis shows massive Glul1 protein synthesis and post-translational modification in consequence of skin injury (right part). A lysate of C57-BL6J retina has been used as an antibody-specific control. Bars indicate means ± SEM obtained from three wounds (n=3) isolated from four individual animals (n=4). ***P* < 0.01; **P* < 0.05 (ANOVA) as compared to control skin (Dunnett´s post-hoc test). C / ctrl: control skin.

**Figure S3. Detection of PKMs phosphorylation and stoichiometry in lysates of murine muscle and murine embryonal fibroblasts.** Pkm1, the solely expressed pyruvate kinase isoform in murine gastrocnemius muscle, is a target for phosphorylation on tyrosine 105 (A). The tetrameric versus dimeric stoichiometry of PKM isoforms as detected by BN-PAGE is shown in (B).

**Figure S4. Temporal expression of VEGF protein during cutaneous wound healing.** Quantification of VEGF_165_ protein expression in murine wounds as assessed by ELISA at the indicated time points after injury. Non-wounded back skin served as a control (ctrl skin). Bars indicate means ± SEM obtained from wounds (n=3) isolated from four individual animals (n=4); ****P* < 0.001; ***P* < 0.01;**P* < 0.05 (ANOVA) as compared to control skin (Dunnett´s post-hoc test). C: control skin.

**Figure S5. Expression pattern of PKM isoforms in normal human epidermal keratinocytes (NHEK) as compared to HaCaT keratinocytes.** (A) Expression of PKM1 or PKM2 mRNAs in NHEK as compared to the expression level of PKM1 (set as 1) or PKM2 (set as 100%) in HaCaT. To assess the relative mRNA expression of PKM2 to PKM1 isoforms in both keratinocytes cells, SYBR signal has been normalized to the length of the amplicons. Bars indicate means ± SD obtained from three independent experiments (n=3) performed in triplicate; ****P* < 0.001; ***P* < 0.01 (unpaired Student's *t*-test). (B) Immunoblot analysis of PKM1 and PKM2 protein expression in HaCaT and NHEK. The expression of GAPDH was used as a loading control.

**Figure S6. Induction of HIF-1α expression in mouse wounds and HaCaT keratinocytes.** (A) A scattered expression pattern of Hif-1α is detected in the hyperproliferative epithelium (HE) of day 1 to 5 mouse wounds. A full wound section is shown at the left side (scale bars: 500 µm) and the marked region is shown in the left part (day 3 scale bars: 200 µm; days 1 and 5 scale bars: 100 µm; day 5 below: scale bar: 50 µm). HE are marked by a dashed yellow line. GT, granulation tissue; SC, scab. Vegf staining is mainly restrained to keratinocytes adjacent to the wound edge. (B) Induction of HIF-1α in HaCaT keratinocytes depends on the hypoxia signal and is not altered by growth factors. Untreated (Mock) or EGF (30 ng/ml) treated HaCaT cells were supplemented by hypoxia mimicking agent CoCl2 (200 µM) in the presence or absence of 1 mM DMOG for 2 hours and harvested in Triton X-100 lysis buffer. 50 µg of total cell lysates were analyzed for the expression of HIF-1α by immunoblot. The expression of β-actin was used as a loading control.

**Figure S7. Pharmacological activation of PKM2 does not alter growth factor induced production of VEGF by HaCaT keratinocytes.** Quiescent HaCaT keratinocytes were treated with 50 µM or 100 µM TEPP-46 to promote PKM2- tetramerization, or remained untreated with medium containing DMSO alone prior to stimulation by EGF. Cells were treated by EGF (30 ng/ml) for 4 hours and VEGF mRNA was quantified as compared to RPLPO housekeeping gene by RT-PCR (A). The release of VEGF protein in culture supernatants of HaCaT cells stimulated by EGF for the indicated time points was assessed by ELISA. VEGF protein is expressed as pg/ml supernatant (B). Bars in (A) and (B) indicate means ±SD obtained from three independent cell culture experiments (n=3) performed in triplicate; metabolic activation of PKM2 does not modify the production of VEGF mRNA or protein induced by EGF (one way ANOVA with Bonferroni’s post-hoc test). The activation of PKM2-dimer and tetramer formation by TEPP-46 in this experimental setting was confirmed by BN-PAGE of 25 µg HaCaT protein-lysate as shown in (C).

**Figure S8. The Silencing of PKM2** **dysregulates inflammatory cytokine-induced activation of STAT3 and translational regulators.** HaCaT keratinocytes were transfected with PKM2-specific siRNA (si156) or control siRNA (siCtrl), and stimulated after 48 hours by 20 ng/ml IL-6 (left) or 20 ng/ml IL-22 (right) for the indicated time points. 25 µg of cell lysates were analyzed by immunoblot to assess the phosphorylation state of STAT3 (Tyr_705_), eIF2α (Ser_51_), and 4E*-*BP1 (Ser_65_). The expression level of the corresponding total protein was used as a loading control. The silencing of PKM2 expression was verified by immunostaining of PKM2 and PKM1 proteins (first and second blot of each panel, respectively). Ponceau S staining (PoncS, left and right panels last blots) show the decrease of a 60 kDa band (black downward arrowheads), subsequent to transfection with si156 (white upward arrow heads) (at the bottom of each panel). A representative blot from three (n=3) independent experiments is shown.

**Figure S9. IL-6 enhances EGF induced angiogenic factor VEGF expression in HaCaT keratinocytes.** HaCaT cells remained untreated (Mock) or dosed with either IL-6 (20 ng/ml) or EGF (30 ng/ml) or both for the indicated time points. VEGF mRNA was quantified as compared to the RPLPO housekeeping gene by RT-PCR (left). The expression level of VEGF mRNA in untreated cells was set as 1 and fold of induction of VEGF mRNA relative to Mock was calculated (right). Bars indicate means ± SD obtained from three independent experiments (n=3) performed in triplicate; *P < 0.05 (ANOVA) (Bonferroni’s post-hoc test).
